# Neuroinflammation as a Common Feature of Neurodegenerative Disorders

**DOI:** 10.3389/fphar.2019.01008

**Published:** 2019-09-12

**Authors:** Leonardo Guzman-Martinez, Ricardo B. Maccioni, Víctor Andrade, Leonardo Patricio Navarrete, María Gabriela Pastor, Nicolas Ramos-Escobar

**Affiliations:** ^1^Laboratory of Neuroscience, Faculty of Sciences, University of Chile & International Center for Biomedicine (ICC), Santiago, Chile; ^2^Department of Neurological Sciences, Faculty of Medicine, University of Chile, Santiago, Chile; ^3^Departamento de Biología, Facultad de Química y Biología, Universidad de Santiago de Chile, Santiago, Chile

**Keywords:** Alzheimer’s disease, Parkinson’s disease, tauopathies, neuroinflammation, microglia, astrocytes, proinflammatory cytokines

## Abstract

Neurodegenerative diseases share the fact that they derive from altered proteins that undergo an unfolding process followed by formation of β-structures and a pathological tendency to self-aggregate in neuronal cells. This is a characteristic of tau protein in Alzheimer’s disease and several tauopathies associated with tau unfolding, α-synuclein in Parkinson’s disease, and huntingtin in Huntington disease. Usually, the self-aggregation products are toxic to these cells, and toxicity spreads all over different brain areas. We have postulated that these protein unfolding events are the molecular alterations that trigger several neurodegenerative disorders. Most interestingly, these events occur as a result of neuroinflammatory cascades involving alterations in the cross-talks between glial cells and neurons as a consequence of the activation of microglia and astrocytes. The model we have hypothesized for Alzheimer’s disease involves damage signals that promote glial activation, followed by nuclear factor NF-kβ activation, synthesis, and release of proinflammatory cytokines such as tumor necrosis factor (TNF)-α, interleukin (IL)-1, IL-6, and IL-12 that affect neuronal receptors with an overactivation of protein kinases. These patterns of pathological events can be applied to several neurodegenerative disorders. In this context, the involvement of innate immunity seems to be a major paradigm in the pathogenesis of these diseases. This is an important element for the search for potential therapeutic approaches for all these brain disorders.

## Cross-Talks Between Glial Cells and Neurons and Origins of Alzheimer’s Disease

The German physician Alois Alzheimer discovered, in the beginning of the past century, a neuropsychiatric disorder, with clinical features of a dementia, called Alzheimer’s disease (AD) after him. He analyzed the *postmortem* brain of an elderly woman with cognitive impairment and found anomalous structures which correspond to the intracellular neurofibrillary tangles (NFTs) formed by aggregates of hyperphosphorylated tau protein. These along with the oligomers of β-amyloid (Aβ) peptide became the major hallmarks of this disease. Along with these hallmarks, during many years of research, several factors have been elucidated, neuroinflammation being a key element in the development of the disease. In dementia, one of the most frequent is AD that affects mainly people over 65 years old. Because of the expansion of life expectancy, AD has become a major health problem, with an estimated 50 million people all over the world having it (Bettens et al., 2010). According to the World Health Organization (WHO), AD progressively affects learning and memory as well as mood and behavior, displaying a constantly increasing prevalence and impact (Maccioni, 2012; Guzman-Martinez et al., 2013).

A major constituent of NFTs is a hyperphosphorylated form of the axonal protein tau, whereas a major constituent of senile plaques (SPs) is Aβ protein. SPs are extracellular deposits and correspond to deposition of Aβ peptides, derived from the amyloid precursor protein (AβPP) (Chapman et al., 2002). Aβ is generated by a sequential processing of the AβPP by two proteases and usually exported from the brain to the cerebrospinal fluid (CSF) and local degradation by microglia, the major constituent of the brain’s innate immune system. In principle, microglia can engulf Aβ by phagocytosis (Heneka et al., 2015).

Hyperphosphorylated tau protein originally forms oligomeric structures called paired helical filaments (PHFs); then it turns into NFTs. The deposition of these structures causes loss of synaptic function and finally neuronal death (Giannakopoulos et al., 2003). Evidence supports the toxicity of tau aggregates when they are exported into the extracellular environment, along with being spread all over the brain (Neumann et al., 2011; Andrade et al., 2017). Studies of cell morphology and organelle distribution under tau overexpression show alterations in transport through the axis by motor axonal microtubule-associated proteins (MAPs) (Cambiazo et al., 1995).

On the other hand, in AD pathophysiology, a key event is neuroinflammation in the central nervous system (CNS). Thus, in this review, we will focus on how neuroinflammatory processes are directly related to cognitive impairment and to the neurodegenerative processes, describing yet the implications of the involvement of both astrocytes and microglia in the inflammatory and neuro-immunomodulatory processes (Fernandez et al., 2008; Morales et al., 2010; Maccioni, 2011; Neumann et al., 2011). The microglial cells regulate the innate immune functions of astrocytes, under both physiological and pathological conditions; the inflammatory factors released by activated microglia can induce transduction of intracellular signals in astrocytes. On the other hand, the reactive astrocytes release factors that favor changes in the permeability of the blood–brain barrier (BBB), resulting in the recruitment of immune cells in the brain parenchyma. This leads to an amplification of the initial innate immune response. In turn, these reactive astrocytes secrete a wide range of factors, such as neurotrophic factors, growth factors, and cytokines, promoting neuronal survival, neurite growth, and neurogenesis. Both the microglia and the astrocytes release various signaling molecules, establishing an autocrine feedback. The feedback between both types of glial cells generates a close reciprocal modulation for various lesions in the CNS (Jha et al., 2019).

There are several neuroinflammatory factors that are involved in both the onset and the progression of AD. This process depends on the innate immune system which includes microglia and astrocytes (Maccioni et al., 2009). Residues from bacteria, viruses, fungi, abnormal endogenous proteins, iron overload, complement factors, antibodies, cytokines, and chemokines, including toll-like receptors (TLRs) and receptor for advanced glycation end products (RAGE), comprise a large number of damage signals, which represent a danger for homeostasis of the CNS, and participate in microglial action and its activation (Shastri et al., 2013). Under these conditions, microglial cells regulate the expression of different surface markers, such as the major histocompatibility complex II (MHC-II) molecular pattern recognition receptors (PPRs), which produce cytokine proinflammatory drugs such as interleukin (IL) 1 beta (IL-1β), IL-6, IL-12, interferon (IFN) gamma (IFN-γ), and tumor necrosis factor (TNF) alpha (TNF-α). They also synthesize and release short-lived cytotoxic factors, such as superoxide radicals (O_2_^−^), nitric oxide (NO), and reactive oxygen species (ROS) (Meda et al., 2001; Colton and Wilcock, 2010). Therefore, and in relation to the above, microglial cells have an important role in innate immunity and are the main source of proinflammatory factors in the human brain. The microglial activation process depends on phenotypic characteristics and is functionally diverse, because the response depends on the type, intensity, and context of the stimulus that generates it. The factors that affect microglia can also generate neuroprotection. Under pathological conditions, neurotoxicity will be expressed, due to the breakdown of the delicate balance between neurotoxic and neuroprotective effects.

Microglial cells exhibit ramified processes having high motility and allowing a dynamic and continual survey of the healthy brain as observed by using *in vivo* two-photon imaging (Nimmerjahn et al., 2005). They sample, detect, and eliminate debris or apoptotic neurons by phagocytosis, but this ability is considerably decreased in a proinflammatory context (Koenigsknecht-Talboo and Landreth, 2005). Microglia is involved in multiple processes such as neurogenesis, synapse elimination in a complement-dependent manner, or synapse plasticity (Paolicelli et al., 2014). The involvement of microglia in AD pathogenesis was studied in the light of the Aβ (Guillot-Sestier et al., 2015; Heneka et al., 2015) and also in the context of tau oligomerization (Maccioni et al., 2009; Morales et al., 2010; Maccioni, 2012; Morales et al., 2014).

Another key factor is the accumulation of monocytes and microglia around blood vessels, due to the CCL2 chemoattractant protein and its affinity receptor CCR2. Studies showed that removal of the receptor increases the microglia accumulation phenomena, possibly through recruitment of mononuclear phagocytes and bone marrow, which promotes the deposition of perivascular Aβ (Ransohoff, 2016b). Care should be taken, since most experiments of circulating monocytes include conditions in which the BBB is open by irradiation procedures in AD, leading to controversy. Interestingly, the reduction of monocyte infiltration following ccr2 deficiency has been involved in tau hyperphosphorylation in traumatic brain injury (TBI) (Ransohoff, 2016b).

It is known that Aβ oligomers induce the activation of microglia through oligomers-surface receptors such as TLRs, being part of a physiological duty to eliminate them *via* phagocytosis (Walter et al., 2007). Certain receptors are associated with the reduction of microglial Aβ phagocytic capacity like triggering receptors expressed in myeloid cells 2 (*TREM*2), whose specific missense mutations increase the risk of AD (Jonsson et al., 2013; Parhizkar et al., 2019). Deficiencies in receptor CX3CR1, a chemokine CX3CL1 microglial receptor which partly mediates the infiltration of monocytes, induce overexpression or activation of microglia and tau hyperphosphorylation (Maphis et al., 2015), increased CD33 with a specific single-nucleotide polymorphism (SNP) in the promoter which inhibits immune response promoting Aβ_1–42_ accumulation (Griciuc et al., 2013), B3 domain-containing transcription factor ABI3 (*ABI3*) (Sims et al., 2017), and several other factors.

On the other hand, some variants in phospholipase C gamma 2 (*PLCG2*) have a positive outcome for AD, reducing the late onset of the disease (Sims et al., 2017). Friedman et al. (2018) determined the gene expression profile associated with neurodegeneration, where 75% of these genes are linked with gene ontology (GO) related to plasmatic membrane. Altogether, this information and the effect of mutations in several receptors and other plasma membrane proteins suggest that changes depend on the interaction with the environment. The paper of Keren-Shaul et al. (2017) describes a new kind of microglia, the disease-associated microglia (DAM) that only gets expressed in AD. The study determined several gene modules. In DAM, a neurodegeneration gene core is expressed. Other modules include the IFN gene. In AD animal models, there are abundant cells that express the IFN module. The DAM gene expression changes as follows: there is downregulation of homeostatic genes like CX3CR1 and upregulation of genes associated with the disease like Apoε and phagocytic genes for plaque clearance. Every microglia has promoters and enhancers associated with DAMs, indicating that these stage changes might pass through an epigenomic change. They also discovered the three stages of microglia: homeostatic, intermediated, and finally DAM stage through an unknown Trem2-independent mechanism. In microglia gene expression modules, lipopolysaccharide (LPS) and neutrophil/monocyte are exacerbated, suggesting that inflammation and infiltration elements are involved in the neurodegenerative disease (ND).

Along with microglia, astrocytes are involved in the neuroinflammation process. Astrocytes have roles in metabolic regulation, neuronal scaffold, and synaptogenesis. In addition, there is a close contact with microglia and blood vessels in BBB (Morales et al., 2014). It also participates in the clearance of Aβ, by enzyme secretion (Mulder et al., 2012), and APOε from the ε2 allele is considered a protective factor. (Koistinaho et al., 2004). Like microglia, astrocytes also surround Aβ plaques (Medeiros and LaFerla, 2013), turning into an activated phase. Calcium deregulation, expression of the APOε4 allele, gives rise to APOε4 activity, which does not affect the synthesis of Aβ but does increase the deposition of the same, meaning a defect in the Aβ clearance (Holtzman et al., 2000). The astrocytes can be activated through a pathway involving NF-κB, to release a C3 complement which binds to the C3aR receptor, inducing neuronal damage (Lian et al., 2015), along with soluble CD40, which binds to microglia and induces the release of TNF-α and other proinflammatory cytokines (Frankola et al., 2011). In neuroinflammation, astrocytes also contribute to NO toxicity, by expressing inducible NO synthase (iNOS) (Phillips et al., 2014). Besides, there is overexpression of the glial fibrillary acidic protein (GFAP), a protein essential in the astrocyte cytoskeleton, related to astrocyte activation (Hol and Pekny, 2015). In the tauopathy context, Aβ can bind to the calcium sensing receptor (CaSR) in astrocytes, which triggers signaling pathways involved in the production and release of phosphorylated tau (Chiarini et al., 2017).

## Neuroinflammation in AD

Neuroinflammation is a process related with the onset of several neurodegenerative disorders and it is an important contributor to AD pathogenesis and progression. Several damage signals appear to induce neuroinflammation, such as trauma, infection, oxidative agents, redox iron, oligomers of tau, and Aβ. In effect, neuroinflammation is responsible for an abnormal secretion of proinflammatory cytokines that trigger signaling pathways that activate brain tau hyperphosphorylation in residues that are not modified under normal physiological conditions. Indeed, evidence exists that AD pathogenesis is not restricted to the neuronal compartment but includes strong interactions with immunological cells in the brain such as astrocytes, microglia, and infiltrating immune cells from the periphery, which could contribute to the modification of the process of neuroinflammation and neurodegeneration in AD brains. In this context, this is where our theory of neuroimmunomodulation plays an important role and focuses on the link between neuronal damage and brain inflammatory process, mediated by the progressive activation of astrocytes and microglial cells with the consequent overproduction of proinflammatory agents (Maccioni et al., 2009). Despite clinical and pathological differences, increasing experimental evidence indicates that neuroinflammatory events lead to tau protein misfolding (Cortes et al., 2018).

The participation of the innate immune system in disease progression has shown a harmful bidirectional connection with regard to tau pathology. It is known that the tau protein belongs to the family of MAPs and is expressed mainly by neurons with preferential axonal localization. It has been observed that tau *in vitro* promotes the polymerization of tubulin and decreases the transition rate between the phases of growth and contraction, generating a stable but dynamic state in microtubules (Weingarten et al., 1975; Drechsel et al., 1992).

Tau is found mainly in axons, but a small amount is distributed physiologically in dendrites. The postsynaptic function of tau is not yet well defined, but it may be involved in synaptic plasticity. On the other hand, in addition to axons and dendrites, a nuclear function of tau (Citron, 2010) has been discovered, which could be regulating transcriptional activity and maintaining DNA/RNA integrity under physiological and stress conditions (Weingarten et al., 1975; Violet et al., 2014).

The tau structure corresponds to a hierarchical phosphorylation process in which different sites modulate the conformation of the protein, promoting the action of secondary kinases. In AD, different sites are phosphorylated earlier than others, leading to the creation of new epitopes. This sequential process has been studied by the use of antibodies such us AT100, whose epitopes in PHFs only appear after successive phosphorylation of residues Thr212 and Ser214, by glycogen synthase kinase (GSK)-3β and protein kinase A (PKA) along with Ser199, Ser202, Ser208, and Thr205 (Bussiere et al., 1999; Malia et al., 2016).

It was also shown that the expression of tau by microglial cells promotes its activation (Wang et al., 2013). Overall, the exact pathway leading to phosphorylation of tau remains poorly defined, but subsequent structural changes induce its detachment from the microtubules and produce higher levels of soluble free tau. Before the formation of NFTs, the hyperphosphorylation of tau favors a dynamic and progressive self-assembly of tau in oligomeric forms and insoluble materials such as PHFs throughout the disease with different degrees of neurotoxicity (Braak and Braak, 1991).

## Neuroinflammation in Several Tauopathies

### Neuroinflammation in the Context of Tau and Tauopathies

These neurodegenerative disorders (tauopathies) do not have a defined clinical, biochemical, and morphological characteristic, like other diseases. Neurodegenerative disorders are distinguished by accumulation of misfolded proteins, such as α-synuclein (α-syn) protein in Parkinson’s disease (PD) and tau protein in AD (Cortes et al., 2018). An important group of degenerative diseases are the so-called tauopathies, which consist in the pathological accumulation of tau protein in intracellular fibrillary aggregates. The spectrum of tauopathies covers a large number of disorders such as progressive supranuclear palsy (PSP), corticobasal degeneration (CBD), frontotemporal dementia (FTD), FTD and parkinsonism linked to chromosome 17, chronic traumatic encephalopathy, and argyrophilic grain disease (Spillantini and Goedert, 2013; Arendt et al., 2016). On the other hand, this last pathology is identified by atrophy of the ambient gyrus and presence of argyrophilic and 4R-tau immunoreactive grains in medial temporal-lobe structures (Tolnay and Clavaguera, 2004). On the other hand, AD is considered a secondary tauopathy because it also presents aggregates of Aβ (SPs) (Musiek and Holtzman, 2015). On the other hand, the pathological aggregates of tau protein, cells of the cerebral immune system, such as activated astrocytes and microglia, are other common pathological features of tauopathies (Wyss-Coray and Mucke, 2002; Ransohoff, 2016a). The existence of neuroinflammatory processes exacerbated in various tau pathologies, known as the *theory of neuroimmunomodulation*, was initially described in AD by Dr. Maccioni’s group, in which the bases of the molecular cascades associated with these events were laid (Maccioni et al., 2009). Recent studies have discovered multiple mechanisms by which an overstimulation of glial cells causes a harmful neuroinflammation that would influence the tau pathology and accelerate the neurodegenerative processes. The chronic activation of glial cells alters the physiological function of the tau protein, inducing the activation of enzymes that phosphorylate tau, such as the enzymes CDK5 and GSK-3β, giving way to the formation of NFTs, thus decreasing the neuronal capacity (Wyss-Coray and Mucke, 2002; Lull and Block, 2010; Ransohoff, 2016a). In addition, glial cells can also contribute physically to the spread of tau pathology (Asai et al., 2015). In turn, the glial cells are also positively fed back by the tau pathology, since the degenerating neurons and their axons and dendrites release aggregated and toxic tau species, generating a constant neuroinflammatory cycle (Morales et al., 2010; Morales et al., 2013; Cortes et al., 2018).

As indicated, activated microglia release proinflammatory cytokines to their cell environment, among which we can highlight IL-1β, IL-6, IL-12, IFN-γ, and TNF-α. In turn, they can also produce ROS and NO, among others that may be characteristic of neurodegenerative disorders (Wang et al., 2015). On the other hand, the astrocytes are the main and most numerous of the glial cells; they are fundamental to supporting the function and health of the neuronal cells. In turn, astrogliosis can also be considered as an important factor in chronic neuroinflammation, affecting considerably the neuron and its integrity (Sofroniew and Vinters, 2010). As microglia, astrocytes also synthesize and secrete proinflammatory cytokines. In addition, experimental evidence has indicated that ILs such as IL-1β, TNF-α, IL-6, and C1q (secreted by the microglia) coactivate the astrocytes, resulting in neuronal dysfunction and ultimately death (Jacobs and Tavitian, 2012). In contrast, in PSP, only IL-1β increased significantly in the *substantia nigra* and in the *subthalamic nucleus* (Fernandez-Botran et al., 2011; Lopez Gonzalez et al., 2016).

Currently, there is an increase in the evidence that the pathological activation of both microglia and astrocytes causes chronic neuroinflammation in patients with tauopathies, negatively affecting the progression of the disease, although the first signs of neuroinflammation considered reactive gliosis in tauopathies and other NDs (Leyns and Holtzman, 2017).

### Frontotemporal Dementia

More than a new pathological entity is the redefinition of the classic Pick’s disease. The term FTD is used to mean all those primary degenerative processes of the anterior portion of the brain, characterized by their clinical manifestations, neuroimaging findings, and histopathological elements, which are of particular importance for psychiatry, due to the tendency of patients to present behavioural disorders, being a frequent cause of dementia (Neary et al., 1988). FTD is a heterogeneous syndrome that involves several disorders that originate mainly in the frontal lobe and temporal areas of the human brain. These alterations can cause problems in language and motor and behavioural disorders (Neary et al., 1998; Olney et al., 2017). In terms of prevalence, after AD, FTD is considered the second most important type of neurodegenerative disorder (Knopman and Roberts, 2011; Hughes et al., 2015). The onset of FTD is between 45 and 65 years of age, with a survival range of between 2 and 20 years, averaging 8 years. In turn, demographic data indicate that the distribution of this disease is similar among men and women (Hodges et al., 2003; Onyike and Diehl-Schmid, 2013; Coyle-Gilchrist et al., 2016), with a prevalence between 15 and 22 people per 100,000 inhabitants, varying according to the age of onset (Irwin et al., 2015). On the other hand, FTD associated with pathological tau represents 36–50% of all cases of FTD (Bang et al., 2015). There are different subtypes of FTD, which are directly related to an existing clinical classification, establishing the following main forms of FTD: the behavior variant of FTD (bvFTD), the non-fluid variant (nfFTD), and the semantic variant (svFTD).

Both nfFTD and svFTD are classified as primary progressive aphasia, since they mainly affect language functions, according to the criteria of clinical diagnosis. The type of FTD that presents the greatest insight is bvFTD, which covers about 60% of cases (Rascovsky et al., 2011; Onyike and Diehl-Schmid, 2013; Olney et al., 2017). Likewise, other alterations in the FTD category are related to motor neuron disorder (MNFTD), PSP-FTD, and corticobasal syndrome (CBS) (Wang and Mandelkow, 2015; Olney et al., 2017). In the same context, frontotemporal lobar degeneration (FTLD) is based on the neuropathological alterations that occur in the frontal and temporal lobes. Different types of FTD are determined by protein aggregates. In this way, the protein aggregates associated with FTD are the following: (i) tau protein (FTLD-tau); (ii) ubiquitin proteasome system (FTLD-UPS); (iii) transactive response of the DNA binding protein (FTLD-TDP); and (iv) fused in sarcoma, Ewing sarcoma, and TAF15 protein family (FTLD-FET) (Mackenzie et al., 2010; Irwin et al., 2015; Olney et al., 2017). For the purpose of this review, we focus on FTDs caused by pathological inclusions of tau, which correspond to approximately half of FTDs, whose histopathological hallmark corresponds to aggregates of tau protein in both neurons and glial cells (Sieben et al., 2012). These include Pick’s disease (PiD-FTD), CB, PSP-FTD, and other rare FTDs, described as globular glial tauopathies and argyrophilic grain disease, which are included in former classifications (Mackenzie et al., 2010; Ghetti et al., 2015; Olney et al., 2017). The pathologies described previously are principally characterized by repetitions of 4R-tau sequences; on the other hand, PiD-FTD are related to 3R-tau aggregates (Ghetti et al., 2015).

Previously, a study reported an increase in the levels of cytokines associated to inflammation, specifically TNF-α and transforming growth factor (TGF)-β in subjects affected by an unspecified form of FTD as compared to healthy people, suggesting a likely interference of inflammatory proteins in the pathogenesis of FTD that was immediately verified by Bellucci et al. (2004); this group reported a considerable rise of cytokines as IL-1 and cyclooxygenase-2 (COX2), both being involved in proinflammatory response; also, studies in transgenic mice with mutation of tau show activation of microglia cells with tau inclusions in the brainstem and spinal cord. Moreover, studies conducted in transgenic mouse with mutation of human tau associated with FTD model reported microgliosis and synaptic disruption, prior to formation of NFT in the hippocampus, suggesting that inflammatory response can carry over to the formation of NFT in FTD (Yoshiyama et al., 2007), in accordance with our theory of neuroimmunomodulation (Fernandez et al., 2008; Rojo et al., 2008; Maccioni et al., 2009). In turn, several trans-models with the FTD tau mutation model have also reported a microglial activation with the subsequent inflammatory process, which further emphasizes that these alterations directly depended on the expression of tau (Wes et al., 2014). In this way, the neuroinflammatory process has been proposed as possible diagnostic tools, through *in vivo* uptake of the microglia, using positron emission tomography (PET) images with the translocator protein (TSPO) ligand [^11^C]-PK11195 in the FTD and other tauopathies (Cagnin et al., 2004; Venneti et al., 2009; Zhang, 2015).

### Progressive Supranuclear Palsy

It is a rare neurodegenerative disorder that is increasing over time. It affects movement, walking, balance, speech, swallowing, vision, mood, behavior, and thinking. One of the classic signs of the disease is the inability to focus and move the eyes correctly, which people can manifest as blurred vision. The prevalence of PSP is 5.8 to 6.5 per 100,000 (Ling, 2016). Like CBD, PSP presents hyperphosphorylated 4R-tau in neurons and glial cells. PSP is defined primarily by tau-positive NFTs, coiled bodies, threads, and tufted astrocytes, in contrast to the ballooned neurons, pre-tangles, threads, and astrocytic plaques that are characteristic of CBD (Yoshida, 2014).

Imaging studies used conventional magnetic resonance; atrophy can be seen at the level of the midbrain and superior cerebellar peduncle. In turn, when using diffusion tensor, the white matter of degeneration can be appreciated, especially in the superior cerebellar peduncles and the superior longitudinal fasciculus in the case of Richardson’s syndrome; dopamine transporter single-photon emission computed tomography imaging shows reduced tracer uptake in the striatum; finally, fluorodeoxyglucose PET may identify focal midbrain hypometabolism (Yoshida, 2014; Ling, 2016; Kovacs, 2017).

The NFTs of tau in subcortical structures are a characteristic sign of PSP. These pathological aggregates of tau are located especially in the subthalamic nucleus, the basal ganglia, and the brainstem. The subcortical NFTs are associated in a variable but characteristic way, with astrocytes in tufts and spiral oligodendroglial bodies, as well as with threads, which present immunoreactivity for the isoform of tau 4 repetitions (4R-tau). Studies conducted by Williams et al. (2007) identified that certain brain regions are affected by these pathological tau aggregates. In this context, the pallido-luyso-nigral system is affected early, followed by the basal ganglia, the pontine nuclei, and the dentate nucleus; then the frontal and parietal lobes; and finally other neocortical areas and cerebellar structures (Williams et al., 2007; Kovacs, 2017). When doing comparative studies between CBD and PSP, a greater amount of pathological tau is observed in neurons in the anterior brain in CBD, whereas in PSP the structures of the posterior brain are mainly affected (Dickson, 1999). In addition, the different astroglial pathology associated with the pathological tau protein, associated with the presence of subcortical NFTs in the PSP, facilitates its neuropathological differentiation (Clavaguera et al., 2013).

### Corticobasal Degeneration

This disease, a rare and progressive neurodegenerative disorder that affects about 4.9 to 7.3 per 100,000 of the population (Mahapatra et al., 2004), is characterized pathophysiologically by neuronal loss, asymmetric frontoparietal cortical atrophy, gliosis, and swollen achromatic neuronal cell bodies (Rebeiz et al., 1968; Gibb et al., 1989). However, the exact cause of CBD still remains unknown, and although CBD is considered a sporadic disease, there have been some reports of families with pathology similar to CBD (Uchihara and Nakayama, 2006) or mutations in tau protein, a gene linked to pathological findings similar to CBD (Mirra et al., 1999; Spillantini et al., 2000). The average age at which the symptoms begin to manifest in the affected subjects, due to this disease, is close to 62 ± 7 years. The average survival can vary from 2 to 13 years (Rinne et al., 1994c; Schneider et al., 1997; Wenning et al., 1998; Josephs et al., 2006a). The youngest case, according to the pathological confirmation, began at age 45 (Wenning et al., 1998). Demographic studies indicate a higher incidence of this disease in women compared to men of the same age (Rinne et al., 1994c).

CBD can be characterized by the following signs and symptoms: action and postural tremor, resting tremor, bradykinesia, myoclonus, ideomotor apraxia, exotic extremity phenomena, extremity dystonia, gait deterioration, dysarthria, aphasia, speech apraxia, and/or dementia; these symptoms can be presented, in turn, in combination (Wenning et al., 1998). In this disease, cognitive deterioration is more affected in the area of speech and language (Josephs, 2010) and, in a lesser extent, visuospatial and perceptual deficits (Tang-Wai et al., 2003a; Bak et al., 2006). The CBD is molecularly characterized by deposits of hyperphosphorylated 4R-tau protein; this makes it possible to differentiate it from other tauopathies, which consist mainly of 3R-tau or a mixture of 3R- and 4R-tau (Trojanowski and Dickson, 2001). Brain neurons with CBD react positively with antibodies generated against ubiquitin, hyperphosphorylated tau, phosphorylated neurofilament protein, alpha-B crystal, and synaptophysin. In addition, aggregates of Aβ have been found in some cases of CBD, which are similar to those found in AD (Armstrong, 2015).

Neuropathological CBD alters the anatomical pathways associated with movement control: (i) striatum and (ii) *substantia nigra*, which usually presents with loss of pigmented neurons. In turn, it also presents at the neuronal level cytoplasmic inclusions and neuronal bodies with greater volume, compared with normal neuronal cells, also presenting glial pathology that affects both oligodendroglia and astrocytes (Armstrong et al., 2000). These damages are especially complex in the posterior frontal area anterior to the precentral gyrus, but they are lesser in the primary motor area (Tsuchiya et al., 1997). These neuronal hallmarks of CBD are found in several brain regions, including the superior temporal gyrus, the frontal cortex, the motor cortex, the brainstem tegmentum, the basal ganglia, the thalamus, and the amygdala (Halliday et al., 1995; Matsumoto et al., 1996).

The experimental evidence shows that the dysregulation of proinflammatory cytokines is one of the most harmful factors in tauopathies. Several investigations have been carried out, using neuroimaging technologies by means of PET, in order to examine more deeply the neuroinflammatory events in the neurodegenerative process. These studies have been used with markers that bind to the TSPO, which is expressed by glial cells (microglia and astrocytes) and other cells of the immune system infiltrating the brain. These findings have shown that the TSPO signal increases proportionally with the activation of microglia in various tauopathies, which include AD, PSP, FTD, and FTDP-17 (Maeda et al., 2011; Zhang, 2015), as well as other NDs, and in models of brain injuries such as FTD, PD, stroke, and traumatic brain injuries (Wu et al., 2013).

The overexpression of IL-1β, TNF-α, and IL-6 has one positive feedback, which generates a cascade that leads to an increase in the hyperphosphorylation of the tau protein, reducing the markers of synapses and finally leading to degeneration and neuronal death (Morales et al., 2014). Currently, less information is available about the levels of cytokine transcription in other less common tauopathies; However, the histological characteristics of activated glial cells are a common characteristic of tau protein aggregates (Morales et al., 2010; Leyns and Holtzman, 2017; Cortes et al., 2018).

**Figure 1:**
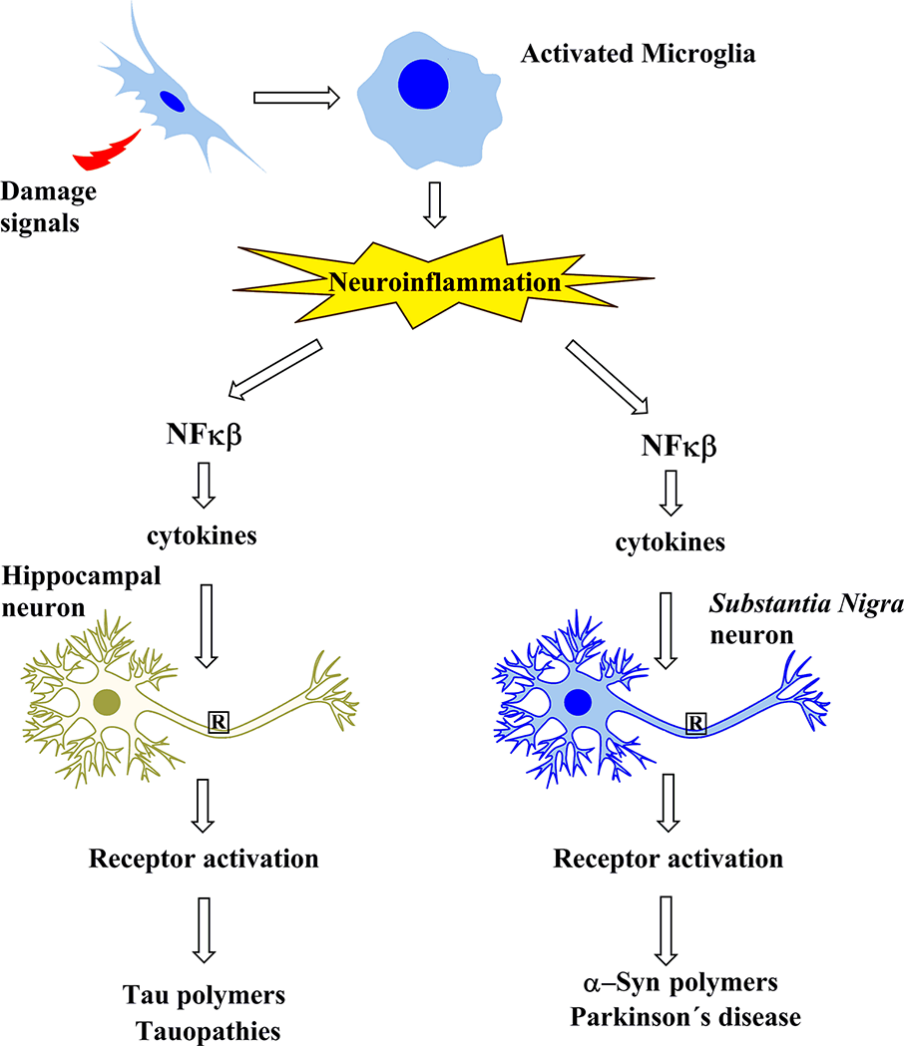
Schematic representation showing that the protein aggregates involved in Alzheimer’s disease and in tauopathies, as well as those of Parkinson’s disease, share a common cascade of molecular signaling as a result of the activation of microglia by signals of damage.

In summary, tauopathies including AD involve the neuroinflammatory cascade that leads to tau modifications and subsequent oligomerization. However, in PD, the neuroinflammatory cascade seems to trigger α-syn oligomerization, an event that is critical in PD (**Figure 1**).

## Future Directions: Search for Therapeutic Targets Based on the Neuroimmunological Model

### Alzheimer Disease

According to the information collected, the microglial cells become one of the points of convergence in the development of the neuroinflammatory process, since not only did the activation process and the changes that occur determine this condition, but it was also shown that a permanent activation can increase the permeability of the BBB and promote an increase in macrophage infiltration peripherals These alterations can complicate the vicinity of the damaged area and contribute to neuronal dysfunction, thus accelerating the neurodegenerative process (Zarrouk et al., 2018).

Within the various factors that modulate the phenotypes activated by the glia, the APO immunomodulatory genotype and the recently identified AD genes can be included. Another important factor to mention is normal aging, which is also associated with the chronic activation of the glia (Zverova et al., 2018) and the factors induced by lesions depending on the focal stage. Context-dependent responses must be expected for nonsteroidal anti-inflammatory drugs (NSAIDs), which act as inhibitors of cyclooxygenase by reducing the concentrations of prostaglandin products, in particular PGE2, by tapping PTGER1–4 receptors and producing very different results. One can highlight the activation of PTGER2, which is predominantly dedicated to proinflammatory neurotoxic pathways, whereas the ligands of PTGER4 have been found to produce anti-inflammatory and neuroprotective effects (Salmon et al., 2015; Alzheimer’s Association, 2018). Thus, NSAIDs that inhibit conventional cyclooxygenase could cause a blockage of the incipient pathogenesis of AD driven by inflammation in the early stages. In addition, these NSAIDs may have adverse effects in advanced disease, mainly by restriction of resolution and interference with phagocytic clearance of Aβ and extracellular tau aggregates (McKee et al., 2008).

Recent findings suggest for anti-Aβ immunotherapy that stage-dependent efficacy also stimulates microglial phagocytosis of Aβ, and the potential benefits could be seen only with early intervention. A remarkable argument is that aging or inflammation induced by Aβ or by lesions initiates tauopathy, leading to neurodegeneration and subsequent clinical decline. Therefore, possible explanations for the failure of immunotherapy to treat established dementia include an inability to stop the spread of fully established and planted tauopathy and to rescue deficits caused by the loss of neurons. Whatever the explanations for the failures of previous NSAID trials, based on new and convincing genetic evidence of the causal role of innate immunity in AD risk, new trials with longer and earlier interventions and alternative approaches to treatment are warranted.

There is evidence that neuroinflammation could drive the pathogenic process in AD. In this context, it should be noted that the brain can no longer be seen as an organ with immune privileges, and advances in immunology must be integrated into the known pathological pathways of various neurodegenerative disorders. Ligand–receptor interactions in the CNS microenvironment, which keep the microglia under strict control in the healthy brain, may be disturbed in chronic ND, although it is not yet clear when and how this occurs in AD. While it is true that the simple idea of activated microglia has been useful, it has undoubtedly prevented the understanding and recognition of the diversity of microglial phenotypes and the extraordinary plasticity of these cells. An important approach that future studies should have should be to better understand the individual contributions of microglia and other cell types to the neuroinflammatory response during the course of AD (Maccioni, 2012; Guzman-Martinez et al., 2013; Morales et al., 2013; Morales et al., 2014).

Innate immune cells of the brain can respond rapidly to systemic events, and these responses are further accentuated in the stages of aging and the diseased brain. In future studies, the effect of systemic comorbidities of AD (such as diabetes and hypertension), associated systemic inflammation, and aging as an important risk factor for AD should be considered in order to understand and exploit the immunological processes associated with AD. The recognition that the modification of the immune system contributes to the pathogenesis of chronic NDs could open several pathways in potential treatments to delay its onset and progression (Morales et al., 2014; Andrade et al., 2017; Cortes et al., 2018).

On the other hand, to date, treatments for AD have been have established based on the nature of the symptomatic AD and, in most cases, are used to counteract the disturbance produced in the level of the neurotransmitters involved in this disease. Therapies based on tau protein suggest that this protein is an interesting target, because the formation of PHFs constitute a critical event in the neurodegenerative process.

An important research suggests that the anti-inflammatory activity may be controlled with a natural formula which contains the Andean compound, a natural product endemic to the north of Chile, and vitamins of the B complex (i.e., B6, B9, and B12), called BrainUp-10®. Interestingly, according to studies done by Cornejo et al. (2011), the active principal of this compound, fulvic acid, is able to block auto-aggregation of tau *in vitro* that inhibits the length and morphology of the PHFs generated. Indeed, this compound can disassemble the preformed PHFs and oligomers and tau species released into the extracellular environment. This natural compound is a potent anti-inflammatory substance and a biologically safe nutraceutical. In a clinical trial pilot, patients with AD who underwent treatment with this compound showed less tendency towards cognitive deterioration, in addition to a reduction in neuropsychological symptoms and less neuropsychiatric stress for caregivers of patients. The Andean compound is a complex mixture of humic substances generated by decomposition of plant material through thousands of years (Carrasco-Gallardo et al., 2012). Currently, there are therapeutic approaches based on the use of antioxidant and anti-inflammatory nutraceutical compounds, a multi-target therapy which appears to have benefits as compared with the mono-target approach using drugs, thus contributing to the health and quality of life of AD patients. Studies based on this type of natural compounds have been growing, as well as the search for natural antioxidant compounds with a strong anti-inflammatory activity and the ability to cross the BBB. Therefore, an important strategy to prevent brain damage is based on changes in lifestyles, diet, and science-based nutraceuticals, among other multiple factors. However, there is a need for further investigations, in order to medically validate this natural approach (Andrade et al., 2017; Cortes et al., 2018).

### Treatments for Tauopathies

Drugs such as cholinesterase inhibitors and *N*-methyl-d-aspartate receptor antagonist are being used to treat cognitive problems. On the other hand, speech and physical therapies have shown positive effects on patients with aphasia syndromes and alterations in motor function, respectively. Serotonin reuptake inhibitors (SSRIs) have been used as a therapy in cases of apathy and depression (Halliday et al., 1995). In addition, specifically, medicaments such as sertraline, paroxetine, and fluvoxamine, which may improve behavioral and psychiatric symptoms but not cognitive impairment, have been selected to treat FTD due to the serotonergic deficit involved (Yoshida, 2014). Citalopram has been reported to lead to an improvement in behavioral symptoms after treatment (Irwin et al., 2015). Acetylcholinesterase inhibitors (AChEIs) are well-established treatments of AD, but until now, there is no report of their efficacy in patients affected by FTD (Kempster et al., 2007). Memantine, which has some effects in advanced AD and neuroprotective characteristics (Ling, 2016), could improve behavioral disorders and enhance metabolic rate in certain brain regions (Williams et al., 2007).

Treatment of cognitive and other symptoms of PSP based on serotonergic drugs has shown to be ineffective, despite its positive effect on depression (Dickson, 1999). Rivastigmine trials in a group of PSP patients reported a mild improvement in cognitive disorders (Garcia-Reitboeck et al., 2013) and had also reported improvement in neuropsychiatric symptoms, but they did not show an effect in cognition (Goedert et al., 2013). Levodopa trials have shown only 20–40% of response in patients affected by PSP, and studies of CBD patients have reported its ineffectiveness because of unresponsiveness (Harper et al., 2008). Actually, some therapeutic strategies are linked to inhibition of tau posttranslational modifications, proteolytic activity, and self-aggregation. The decrease of tau mRNA with antisense oligonucleotides has been reported to decrease harmful tau aggregates, disrupt neuronal loss, and increase life duration of transgenic mice expressing human tau harboring the disease-associated P301S mutation (PS19 mouse model). Furthermore, a study of monkeys has reported a drastic decrease of tau mRNA and protein in the CNS (King et al., 2013). Also, the Food and Drug Administration (FDA) has approved an antisense oligonucleotide directed against mutant survival motor neuron gene 1 (*SMN1*) for treatment of spinal muscular atrophy (Bezard et al., 2013), characterized by alteration of lower motor neurons. Studies of brain injury in mice treated with an antibody directed against phospho-tau (p-tau) reported that it halted formation of tau oligomers and complexes, prevented expansion of harmful tau in neighbor cells, decreased brain atrophy, and regained long-term potentiation (Arendt et al., 2016).

According to the existing relation among tauopathies and insulin resistance, therapies previously utilized for treatment of diabetes approved by the FDA are being tested in preclinical and clinical trials. Insulin supplied intranasally in subjects presenting amnestic mild cognitive disorder or AD has been reported to have cognitive benefits (Jochum et al., 2004), (Musiek and Holtzman, 2015), and thus, this opened the way to a new clinical trial: the Study of Nasal Insulin in the Fight Against Forgetfulness (SNIFF, NCT01767909). This trial is studying the effects of insulin supplied intranasally on cognition and brain atrophy; it is not actually certain how insulin administered by this way affects tau protein (Orr et al., 2017). Liraglutide, a drug which stimulates insulin production, has been shown to prevent and decrease the phosphorylation of tau in a mouse model of type II diabetes (Wyss-Coray and Mucke, 2002) and to decrease phosphorylated tau and improve motor function in a mouse model of tauopathy (hTauP301L); also, there is a report about improvement in motor function on this (Ransohoff, 2016a). Studies on 3xTg-AD mice treated with linagliptin has shown that there is a decrease of tau phosphorylation and improvement in cognition (Asai et al., 2015). Metformin, a suppressor of hepatic glucose production, tested in a neuronal cell model of insulin resistance has been reported to prevent tau phosphorylation (Sarazin et al., 2003). A decrease in pathological tau phosphorylation has been reported in trials with tau transgenic mice, along with an increase in tau cleavage, aggregation, synaptic disruption, and hind limb atrophy (Maccioni et al., 2009).

Immunotherapy-based antibodies against tau and active immunizations targeting pathogenic tau oligomers are actually in clinical trials (Riederer et al., 2003). These would prevent intercellular tau spread. For instance, tau vaccine (AADvac1) has been reported to have favorable safety and immunogenicity outcomes (Bussiere et al., 2003), and antibodies against tau have been shown to be assimilated by neurons in *ex vivo* cultured brain slices *in vivo* (Grant et al., 1997; Kovari et al., 2003; Josephs et al., 2006b; Yu et al., 2016), and tau linked to an antibody may be removed by a pathway implying lysosome (Krishnamurthy et al., 2011; Collin et al., 2014). Rapamycin, a mammalian target of rapamycin (mTOR) inhibitor, upregulates autophagy, thus decreasing aberrant tau and improving cognition in multiple mouse models (Laihinen et al., 1994; Lee et al., 1994; Rinne et al., 1994a; Rinne et al., 1994c), and prevents neuronal death in tau transgenic *Drosophila* (Rinne et al., 1994b). Studies conducted by Salonen et al (1994) with transgenic mice of human tau P301S, observed that rapamycin decreases both phosphorylation of tau and its aggregate state when it is delivered at different stages of the disease. In turn, in trials with 3xTg-AD mice, the intake of prophylactic rapamycin notably prevents the formation of aberrant tau deposits (Salonen et al., 1994). Analogs of rapamycin have been reported to increase autophagy, decrease p-tau and NFTs, and improve cognition in studies with tau transgenic mice (Tang-Wai et al., 2003b; Bak et al., 2006). The natural compound wogonin that inhibits mTOR demonstrated a reduction of p-tau in cultured cells (Tang-Wai et al., 2003a). In *Drosophila*, a decrease of tau-induced neurotoxicity has been achieved by multiple strategies such as heterochromatin loosening, genetic reversal of filamentous actin, nuclear envelope disorder, and decline of oxidative stress (Schmidt et al., 2001; Trojanowski and Dickson, 2001; Fulga et al., 2007; Kandimalla et al., 2014; Armstrong, 2015).

## The Neuroimmune Context of PD

### Neuroimmunology of PD

After AD, the second most common neurodegenerative disorder is PD. This pathology affects the normal movement of the subject, as a consequence of multifactorial factors, such as environmental and genetic factors. The molecular basis is still unclear. The triggering causes have not yet been determined, but it is known that factors such as oligomerization of α-syn, mitochondrial dysfunction, oxidative stress, inflammation, and aging have pathogenic roles in the disease Its main neuropathological marker is the degeneration of neurons which contain neuromelanin in *substantia nigra pars compacta*; this results in loss of dopamine and cytoplasmic protein aggregates, called Lewy bodies (LBs), mainly composed by α-syn filaments (Forno, 1996). α-Syn has 140 amino acids and three regions, a carboxyl end which is negatively charged, an amino terminal end which is charged positively, and a hydrophobic segment at the center, between residues 61 and 90 (considered as the non-amyloid component or NAC). The protein has four tyrosine residues, Tyr39 next to the amino region and Tyr125, Tyr133, and Tyr136 close to the C-terminus. It is also able to bind lipids (Uversky, 2007).

Among people over 50 years, ∼2.0% is affected with PD (Maarouf et al., 2012). The clinical signature consists of motor, cognitive (dementia), neuropsychiatric (depression and anxiety), and autonomic dysfunctions (hypotension and constipation). The motor affections usually present (i) rest tremor, (ii) bradykinesia (impairment in the normal movement, mainly of complex voluntary movements), (iii) postural instability, and (iv) rigidity (Thomas and Beal, 2007). There are a diminished number of dopaminergic neurons in the *substantia nigra*, which consequently ameliorates dopamine in the striatum, promoting dysregulation in the basal ganglia. The previous effects trigger the motor symptoms observed. Briefly, this pathology is considered a ND characterized among the synucleinopathies, which also considers PD with dementia (PDD), LB dementia (DLB), and multiple-system atrophy (MSA). DLB is also able to present parkinsonism, but also hallucinations (mainly visual) and dementia (McKeith et al., 2005). These symptoms make the diagnosis for this disease difficult (McKeith et al., 2005).

### Relationships Between Tauopathies and Synucleinopathies

α-Syn hyperphosphorylation promotes misfolding and oligomerization. α-Syn deposits are ubiquitous in the CNS, commonly at presynaptic neuron terminals. These molecular effects are categorized among synucleinopathies (Golde and Miller, 2009; Uversky, 2009). Synucleinopathies usually share their occurrence with tauopathies and different diseases commonly associated with protein misfolding. Although the etiology of most of the processes involved in these pathologies suggest the effect of misfolded proteins in pathways, also affecting them, they are still unknown (Jellinger, 2010a; Jellinger, 2010b; Kovacs et al., 2010). It seems there is an overlap in both diseases, but they also present different genetic, clinical and pathological characteristics. It is common to find NFTs and LB presence in the brain or in a cell (Arai et al., 2001; Iseki et al., 2003). Their co-occurrence has been previously reported several times by (i) Schneider et al. (2006), who found NFT in the substantia nigra of PD patients with displacement damage, and (ii) Joachim et al. (1987), who found them in the same region in AD patients, Down syndrome, and PD. Furthermore, the presence of p-tau has been noticed in dopaminergic neurons of PD and PDD subjects (Wills et al., 2010). p-Tau has also been seen in striatal neurons of a transgenic model that overexpress human α-syn (Haggerty et al., 2011). Moreover, it was noticed that the phosphorylation of the GSK-3β protein does not happen if the expression of α-syn is silenced (Duka et al., 2006). Finally, these mechanisms considering tau and α-syn can be discussed by this manner: the promotion of α-syn expression leads to its accumulation in the brain, so on, GSK-3β gets phosphorylated, which in turn phosphorylate tau (Duka et al., 2006). NFT start forming because of the increase in p-tau. It is also interesting that almost 60% of AD subjects present LB, in familial and sporadic cases (Arai et al., 2001; Jellinger, 2011). We can consider an aggressive progression of these pathologies and an accelerated cognitive dysfunction because of the presence of synucleinopathies and tauopathies at the same time (Langlais et al., 1993; Olichney et al., 1998; Kraybill et al., 2005). It is suggested that the synergistic interaction between tau, Aβ, α-syn and the activate form of GSK-3β could trigger their misfolding, oligomerization and accumulation (Giasson et al., 2003; Lee et al., 2004).

There has been studies that shown the tau/α-syn binding *in vitro*, which in turn, promote their phosphorylations (Jensen et al., 1999). Tau fibril generation could be induced by α-syn, but also they induce pathological filaments formation between each other when they are co-incubated (Giasson et al., 2003). The tau and α-syn interaction have also been observed *in vivo*, using mice that overexpress Ala53Thr α-syn (A53T SNCA), evidencing aggregation in both proteins (Giasson et al., 2003). Besides, p-tau in Ser396 and Ser202, 396/404, were found in PD cortex synapses (Muntane et al., 2008) and brainstem samples, respectively, in mice models overexpressing A-309P α-syn (Frasier et al., 2005). Moreover, the direct relation of tau and α-syn in these diseases is supported by: (i) hyperphosphorylation of tau as consequence of α-syn effect in the mice model for PD, 1-methyl-4-phenyl-1,2,3,6-tetrahydropyridine (MPTP) (Duka et al., 2006), (ii) α-syn and p-tau presence in NFT and LBs (Shao et al., 2006); (iii) the proteasome promotes the oxidation of α-syn, which in turn triggers recruitment of tau inside oligodendroglial cells in synucleinopathies (Riedel et al., 2009). In addition, it was also observed tau phosphorylation by α-syn in an *in vitro* study, in the residues Ser262 and Ser356 by the PKA (Jensen et al., 1999). As an interesting fact, GSK-3β does not phosphorylate tau in Ser262, meanwhile PKA does not in residues Ser396 and Ser404, suggesting their synergic role in the development of tauopathies mediated by α-syn.

It was reported that α-syn could promote activation of GSK-3β, hyperphosphorylation tau in Thr181, Ser396, and Ser404 (Duka et al., 2006; Duka et al., 2009; Kawakami et al., 2011; Ciaccioli et al., 2013). It seems that this is a consequence of an augmented activity in GSK-3β (Duka et al., 2009; Wills et al., 2011) but also of the generation of a tau, α-syn, and GSK-3β complexes. Although there are more kinases binding to hyperphosphorylated tau and α-syn. Actually, c-Jun N-terminal kinase (JNK) and extracellular signal-regulated kinase (ERK), which also phosphorylate tau in Ser404 and Ser396, were shown by fluorescence intensity distribution analysis (FIDA) to have correlation with the p-tau presence in mice models of PD (α-syn overexpression) (Frasier et al., 2005; Kaul et al., 2011; Oaks et al., 2013). It was also showed that in the presence of inductors for cationic aggregation (Fe^3+^, Al^3+^, or DMSO) co-oligomers of α-syn and tau, and their co-aggregated forms are present at nanomolar concentrations (Nubling et al., 2012). Nonetheless, tau phosphorylation by GSK-3β strongly promotes mixed oligomers appearance (Nubling et al., 2012). Finally, we are demonstrating that α-syn polymerization is promoted by tau and that α-syn induce the same to tau, through the NAC hydrophobic region. In line with this, the main difference of both, is that α-syn aggregates itself, meanwhile tau cannot and needs an inductor (Goedert et al., 1996). Considering all the previous data, we are able to integrate the molecular mechanisms discussed into the neuroimmunomodulation theory (Maccioni et al., 2009) described above, since there are common effectors with the capacity to trigger neuronal damage and death by inflammatory processes.

### Neuroinflammation and Parkinsonism

Following the inflammatory cascade triggered by damage signals, there is synapse impair by several molecular mechanisms. The previous process also generates a positive feedback loop promoting even more damage, mainly mediated by microglial cells (Rojo et al., 2008; Andrade et al., 2017).

Increased permeability of the BBB and neurovascular dysfunction have been associated with severe conditions in PD. This effect could be linked to infiltration of inflammation molecules to the middle brain, microglia activation and dopaminergic neurons death (Collins et al., 2012). The systemic inflammatory response in PD seems to be promoted by peripheral lymphocytes activation and augmented levels of serum cytokines, such as IL-2, IL-6 and TNF-α in PD patients (Collins et al., 2012). However, there is not a general confirmation on the release of proinflammatory cytokines associated with PD. The adaptive immune response could be explained by elevated levels of MHC II in astrocytes of the ventral midbrain and microglia in a mice PD model, after inflammatory processes induction (Martin et al., 2016). Among the different mechanisms which direct microorganisms or non-cerebral immune cells into the brain, one of the recently described are direct vascular channels. They connect the skull bone marrow to the brain surface through the meninges, enabling other cells to travel into this region, which is commonly considered as “aseptic” (Kandimalla et al., 2011b). Leukocytes derived from the bone marrow can trigger inflammatory processes in the tissue they exert their protective function. In this context, it is well established that several brain pathologies, which involved neurodegeneration, commonly present neuroimmune dynamics triggering neuroinflammation, among their paramount action mechanisms (Rojo et al., 2008; Andrade et al., 2017). Since the immune response is also triggered by pathogens, inflammatory processes are elicited as well. This could explain part of the onset and progression of not just PD, but also, several neuronal illness conditions, after their invasion in the brain.

As widely discussed, microglia activation can be triggered by different damage signals including pathogens, toxins, endogenous proteins, products generated by dying neurons, and other toxic agents. The constitutive expression of proinflammatory cytokines, such as IL-2, IL-6, IL-1β, TNF-α, and IFN-γ, the presence of ROS and eicosanoids, were also noticed in *postmortem* patients with PD by cerebral analysis. Moreover, it was seen in serum and CSF *in vivo*, and in animal models for PD. The microglial activation product of neuronal death may lead to a vicious circle of neuroinflammation and neurodegeneration (Collins et al., 2012). Some of these substances released upon degeneration of neurons include aggregates of α-syn, neuromelanin, adenosine triphosphate, and metalloproteinase-3 (MMP-3) (Collins et al., 2012). The activation of microglial cells by the pathological forms of α-syn in PD, DLB and multisystemic atrophy, results in a balance between the elimination of α-syn-mediated phagocytosis through neuronal dysfunction, TLR4 microglia and degeneration of neurons because of proinflammatory cytokines and ROS presence (Bruck et al., 2016). Recently, astrocytes have been also implied in brain degradation of α-syn fibrils, instead of spreading. This mechanism is triggered by the transfer of α-syn to neighbor cells, which is more efficient into astrocytes, which then localize inside lysosomes, where they seem to be degraded (Kandimalla et al., 2011a). Later, since lysosomal dysfunction is a common hallmark of NDs, it was shown that α-syn fibrils are transferred through tunneling nanotubes (TNTs) between neurons inside lysosomes and induce the misfolding/aggregation of the normal soluble protein (Wani et al., 2011). Finally, the stressed lysosomes that function as the vehicle of transfer α-syn fibrils inside TNTs allows, after fibrils escape, the seeding of cytosolic protein, thus explaining the progression of the pathology and highlighting an unsuspected role of lysosomes in this process. (Kandimalla et al., 2013). Thus, and considering also the presence p-tau and NFT in regions affected in PD as discussed before, we are again, able to consider the neuroimmunomodulation theory in the context of inflammation for PD.

The new insights are the gut-brain axis regulation, and its role in the pathogenesis of PD. Gut disorders present another way to induce an immune response and inflammatory processes which could contribute to PD pathology. The most common early manifestations in PD that don’t affect movement or motor skills, are olfactory impairment and constipation. This is shared with the Braak staging system: the enteric nervous system (ENS), α-syn in the dorsal motor nucleus of the vagus nerve, the olfactory bulb and the submandibular gland, since all of them are a gateway to the environment. The neuropathological process leading to the pathology seems to start in the ENS or the olfactory bulb, spreading by the rostrocranial transmission to the *substantia Nigra* and finally to the CNS, suggesting that the environment could be part of the disease onset and development.

For instance, it was shown that the pesticide rotenone can almost completely reproduce the common clinical and pathogenic features of PD after intragastric administration (Kandimalla et al., 2011b). It has been reported that there is an altered intestinal microbiome in patients with PD and that there is an influence of gut microbiota in enteric neuron activity (Chang et al., 2017; Maldonado-Lasuncion et al., 2018; Wei et al., 2018). Alselmi and co-workers evaluated that the gastric coadministration of subthreshold doses of lectins and paraquat reproduces the disease symptoms in rats and its behavioral affections. They used a solution containing paraquat + lectin for administration *via* gastric gavage and then evaluated behavior in the context of PD and gastric dysmotility. Pathological α-syn in the dorsal motor nucleus of the vagus (DMV) and in the *substantia nigra pars compacta* of SNpc neurons was also observed. Besides, nigrovagally evoked gastric motility was affected in the rats which underwent the treatment. This was seen before the onset of PD manifestations, which were improved by l-dopa treatment. They also made a vagotomy, preventing the progression of PD effects and constraining the appearance of pathological α-syn only to enteric neurons. These reports demonstrated that coadministration of these molecules induces a progressive, l-dopa-responsive PD which is preceded by gastric dysmotility (Anselmi et al., 2018). Finally, it is important to notice the role of the microbiota in the CNS, where the host is constantly controlling the maturation and function of the microglial cells (Cosin-Tomas et al., 2018), a reason to consider their effects and imbalances in the subject, which, in the future, could be key in the control, prevention, or even treatment of PD.

Another factor for evaluation is sleep disorders that are common in these diseases, the most common being called rapid eye movement (REM) sleep behavior disorder (RBD), representing between 30% and 46% of the cases for PD (Gagnon et al., 2002) and 50–80% of the cases for DLB (Boeve et al., 2007). Even Donaghy and McKeith (2014) suggest that this symptom could be used as a previous diagnosis for DLB. This is interesting to consider, since previous research has pointed out that REM sleep disturbances could be associated with the formation of aggregated forms of the proteins already discussed, promoting the mechanisms underlying the inflammatory context (Hebert et al., 2008; Leidinger et al., 2013; Nagaraj et al., 2017). As a conclusion, the new pharmacotherapies proposed and their further research for these pathologies, and possible sleep-disruptive effects in PD understanding, are crucial in order to improve the quality of life of the patients.

### Novel Therapies for Parkinson

The pharmacological approaches in PD are the usual modality to treat the pathology. Oral levodopa and a dopamine decarboxylase inhibitor, such as carbidopa, are considered as the best therapeutic agents (Geekiyanage and Chan, 2011). In the immunomodulatory context, several approaches target to diminish the inflammatory response. Recently, the focus has been mainly aimed to the immune signaling from the periphery of the CNS. Williams et al. proposed to target the chemokine receptor type 2 (CCR2) and the MHC OO (MHCII), since this has been previously demonstrated as neuroprotective in rodent models of PD (Martinez and Peplow, 2019). The group evaluated the genetic knockout and RNA silencing of the class II transactivator (CIITA), which coactivates transcriptionally MHCII. Their results provided evidence that CIITA is needed for the induction and infiltration of MHCII in peripheral immune cells by α-syn, in a mice model for PD, presenting it as a novel promising therapeutic target (Benussi et al., 2017). In this context, it is also important in a preventive manner, since it has also been reported that peripheral immune cell recruitment occurs prior to neurodegeneration and microglia; monocytes and macrophages all contribute to MHCII expression in PD (Du et al., 2018). Harms et al. also demonstrate that extravasation of proinflammatory peripheral monocytes into the CNS has a paramount role in neurodegeneration. Using a PD synucleinopathy model, they ended up proposing that peripheral monocytes are targets for PD, as a neuroprotective therapy. They observed that the expression of the full-length human α-syn *in vivo* promotes the infiltration of proinflammatory CCR2+ peripheral monocytes into the *substantia nigra*. Moreover, they found that α-syn-induced monocyte entry can be prevented by the genetic deletion of CCR2, attenuating MHCII expression and ending the degeneration of dopaminergic neurons (Sjodin et al., 2017).

Lots of immunotherapies for the treatment of PD use vaccines with AS or antibodies against it. A variety of procedures for vaccination have evidenced that the induction of regulatory T cells in the periphery protects the animal in PD models. In this context, the formulation glatiramer acetate (Copaxone^®^), which is commonly used for treatment of multiple sclerosis, is presented as a possible candidate because of its capacity to increase the number and the action as suppressors of regulatory T cells (Wani et al., 2014). Several reviewers also considered other approaches in order to diminish the inflammatory context; for instance, transcription factors were proposed in order to address this issue. There are alternatives like this promoting inflammation by effectors such as STAT 3, AP1, NF-κB, and TLRs which are constitutively upregulated in PD, while pathways considered as neuroprotective such as TGF-β, YY1, and mTOR are significantly downregulated in the microglia of patients with PD. Finally, it seems that their regulation could contribute to novel agent generation in order to treat PD, improve patient condition, or prevent the development of the pathology (Pal et al., 2016).

## Author Contributions

LG-M and NR-E prepared the section on tauopathies and their therapy; LN and MP wrote the Alzheimer’s disease section; VA prepared the Parkinson’s disease section; and RM organized the strategy and goals of the paper and coordinated the different sections in the final writing of the text.

## Funding

This study was supported by the CORFO Innova projects (Grant 17ITE2-87685), the International Center for Biomedicine (ICC), and “the Ricardo Benjamin Maccioni Foundation.”

## Conflict of Interest Statement

The authors declare that the research was conducted in the absence of any commercial or financial relationships that could be construed as a potential conflict of interest.

## References

[B1] Andrade V. Guzmán-Martínez L. Pulgar K. V. Maccioni R. B. (2017). “Neuroimmune dynamics in Alzheimer’s disease progression,” in Mechanisms of neuroinflammation. Ed. Abreu G. E. A. (Rijeka: InTech). 10.5772/intechopen.68941

[B2] Anselmi L. Bove C. Coleman F. H. Le K. Subramanian M. P. Venkiteswaran K. (2018). Ingestion of subthreshold doses of environmental toxins induces ascending Parkinsonism in the rat. NPJ Parkinsons Dis. 4, 30. 10.1038/s41531-018-0066-0 30302391 PMC6160447

[B3] Arai Y. Yamazaki M. Mori O. Muramatsu H. Asano G. Katayama Y. (2001). Alpha-synuclein-positive structures in cases with sporadic Alzheimer’s disease: morphology and its relationship to tau aggregation. Brain Res. 888 (2), 287–296. 10.1016/S0006-8993(00)03082-1 11150486

[B4] Arendt T. Stieler J. T. Holzer M. (2016). Tau and tauopathies. Brain Res. Bull. 126 (Pt 3), 238–292. 10.1016/j.brainresbull.2016.08.018 27615390

[B5] Armstrong R. A. (2015). “Corticobasal degeneration and dementia,” in Diet and nutrition in dementia and cognitive decline. Eds. Martin C. R. Preedy V. R. (San Diego: Academic Press), 35–43. 10.1016/B978-0-12-407824-6.00004-5

[B6] Armstrong R. A. Cairns N. J. Lantos P. L. (2000). A quantitative study of the pathological lesions in the neocortex and hippocampus of twelve patients with corticobasal degeneration. Exp. Neurol. 163 (2), 348–356. 10.1006/exnr.2000.7392 10833308

[B7] Asai H. Ikezu S. Tsunoda S. Medalla M. Luebke J. Haydar T. (2015). Depletion of microglia and inhibition of exosome synthesis halt tau propagation. Nat. Neurosci. 18 (11), 1584–1593. 10.1038/nn.4132 26436904 PMC4694577

[B8] Alzheimer’s Association (2018). 2018 Alzheimer’s disease facts and figures. Alzheimers Dement. 14 (3), 367–429. 10.1016/j.jalz.2018.02.001

[B9] Bak T. H. Caine D. Hearn V. C. Hodges J. R. (2006). Visuospatial functions in atypical parkinsonian syndromes. J. Neurol. Neurosurg. Psychiatry 77 (4), 454–456. 10.1136/jnnp.2005.068239 16543521 PMC2077492

[B10] Bang J. Spina S. Miller B. L. (2015). Frontotemporal dementia. Lancet 386 (10004), 1672–1682. 10.1016/S0140-6736(15)00461-4 26595641 PMC5970949

[B11] Bellucci A. Westwood A. J. Ingram E. Casamenti F. Goedert M. Spillantini M. G. (2004). Induction of inflammatory mediators and microglial activation in mice transgenic for mutant human P301S tau protein. Am J Pathol 165, 1643–1652 15509534 10.1016/S0002-9440(10)63421-9 PMC1618683

[B12] Benussi L. Binetti G. Ghidoni R. (2017). Loss of neuroprotective factors in neurodegenerative dementias: the end or the starting point? Front. Neurosci. 11, 672. 10.3389/fnins.2017.00672 29249935 PMC5717017

[B13] Bettens K. Sleegers K. Van Broeckhoven C. (2010). Current status on Alzheimer disease molecular genetics: from past, to present, to future. Hum. Mol. Genet. 19 (R1), R4–R11. 10.1093/hmg/ddq142 20388643 PMC2875058

[B14] Bezard E. Yue Z. Kirik D. Spillantini M. G. (2013). Animal models of Parkinson’s disease: limits and relevance to neuroprotection studies. Mov. Disord. 28 (1), 61–70. 10.1002/mds.25108 22753348 PMC3517687

[B15] Boeve B. F. Silber M. H. Saper C. B. Ferman T. J. Dickson D. W. Parisi J. E. (2007). Pathophysiology of REM sleep behaviour disorder and relevance to neurodegenerative disease. Brain 130 (Pt 11), 2770–2788. 10.1093/brain/awm056 17412731

[B16] Braak H. Braak E. (1991). Neuropathological stageing of Alzheimer-related changes. Acta Neuropathol. 82 (4), 239–259. 10.1007/BF00308809 1759558

[B17] Bruck D. Wenning G. K. Stefanova N. Fellner L. (2016). Glia and alpha-synuclein in neurodegeneration: a complex interaction. Neurobiol. Dis. 85, 262–274. 10.1016/j.nbd.2015.03.003 25766679 PMC4730552

[B18] Bussiere T. Gold G. Kovari E. Giannakopoulos P. Bouras C. Perl D. P. (2003). Stereologic analysis of neurofibrillary tangle formation in prefrontal cortex area 9 in aging and Alzheimer’s disease. Neuroscience 117 (3), 577–592. 10.1016/S0306-4522(02)00942-9 12617964

[B19] Bussiere T. Hof P. R. Mailliot C. Brown C. D. Caillet-Boudin M. L. Perl D. P. (1999). Phosphorylated serine422 on tau proteins is a pathological epitope found in several diseases with neurofibrillary degeneration. Acta Neuropathol. 97 (3), 221–230. 10.1007/s004010050978 10090668

[B20] Cagnin A. Rossor M. Sampson E. L. Mackinnon T. Banati R. B. (2004). *In vivo* detection of microglial activation in frontotemporal dementia. Ann. Neurol. 56 (6), 894–897. 10.1002/ana.20332 15562429

[B21] Cambiazo V. Gonzalez M. Maccioni R. B. (1995). DMAP-85: a tau-like protein from *Drosophila melanogaster* larvae. J. Neurochem. 64 (3), 1288–1297. 10.1046/j.1471-4159.1995.64031288.x 7861162

[B22] Carrasco-Gallardo C. Farias G. A. Fuentes P. Crespo F. Maccioni R. B. (2012). Can nutraceuticals prevent Alzheimer’s disease? Potential therapeutic role of a formulation containing shilajit and complex B vitamins. Arch. Med. Res. 43 (8), 699–704. 10.1016/j.arcmed.2012.10.010 23131823

[B23] Chang W. S. Wang Y. H. Zhu X. T. Wu C. J. (2017). Genome-wide profiling of miRNA and mRNA expression in Alzheimer’s disease. Med. Sci. Monit. 23, 2721–2731. 10.12659/MSM.905064 28578378 PMC5467707

[B24] Chapman M. R. Robinson L. S. Pinkner J. S. Roth R. Heuser J. Hammar M. (2002). Role of *Escherichia coli* curli operons in directing amyloid fiber formation. Science 295 (5556), 851–855. 10.1126/science.1067484 11823641 PMC2838482

[B25] Chiarini A. Armato U. Gardenal E. Gui L. Dal Prà I. (2017). Amyloid β-exposed human astrocytes overproduce phospho-tau and overrelease it within exosomes, effects suppressed by calcilytic NPS 2143—further implications for Alzheimer’s therapy. Front. Neurosci. 11, 217. 10.3389/fnins.2017.00217 28473749 PMC5397492

[B26] Ciaccioli G. Martins A. Rodrigues C. Vieira H. Calado P. (2013). A powerful yeast model to investigate the synergistic interaction of α-synuclein and tau in neurodegeneration. PLoS One 8 (2), e55848. 10.1371/journal.pone.0055848 23393603 PMC3564910

[B27] Citron M. (2010). Alzheimer’s disease: strategies for disease modification. Nat. Rev. Drug Discov. 9 (5), 387–398. 10.1038/nrd2896 20431570

[B28] Clavaguera F. Akatsu H. Fraser G. Crowther R. A. Frank S. Hench J. (2013). Brain homogenates from human tauopathies induce tau inclusions in mouse brain. Proc. Natl. Acad. Sci. 110 (23), 9535–9540. 10.1073/pnas.1301175110 23690619 PMC3677441

[B29] Collin L. Bohrmann B. Gopfert U. Oroszlan-Szovik K. Ozmen L. Gruninger F. (2014). Neuronal uptake of tau/pS422 antibody and reduced progression of tau pathology in a mouse model of Alzheimer's disease. Brain 137 (10), 2834–2846. 10.1093/brain/awu213. 25085375

[B30] Collins L. M. Toulouse A. Connor T. J. Nolan Y. M. (2012). Contributions of central and systemic inflammation to the pathophysiology of Parkinson’s disease. Neuropharmacology 62 (7), 2154–2168. 10.1016/j.neuropharm.2012.01.028 22361232

[B31] Colton C. Wilcock D. M. (2010). Assessing activation states in microglia. CNS Neurol. Disord. Drug Targets 9 (2), 174–191. 10.2174/187152710791012053 20205642

[B32] Cornejo A. Jimenez J. M. Caballero L. Melo F. Maccioni R. B. (2011). Fulvic acid inhibits aggregation and promotes disassembly of tau fibrils associated with Alzheimer’s disease. J. Alzheimers Dis. 27 (1), 143–153. 10.3233/JAD-2011-110623 21785188

[B33] Cortes N. Andrade V. Guzman-Martinez L. Estrella M. Maccioni R. B. (2018). Neuroimmune tau mechanisms: their role in the progression of neuronal degeneration. Int. J. Mol. Sci. 19 (4), 956. 10.3390/ijms19040956 PMC5979395 29570615

[B34] Cosin-Tomas M. Alvarez-Lopez M. J. Companys-Alemany J. Kaliman P. Gonzalez-Castillo C. Ortuno-Sahagun D. (2018). Temporal integrative analysis of mRNA and microRNAs expression profiles and epigenetic alterations in female SAMP8, a model of age-related cognitive decline. Front. Genet. 9, 596. 10.3389/fgene.2018.00596 30619445 PMC6297390

[B35] Coyle-Gilchrist I. T. Dick K. M. Patterson K. Vazquez Rodriquez P. Wehmann E. Wilcox A. (2016). Prevalence, characteristics, and survival of frontotemporal lobar degeneration syndromes. Neurology 86 (18), 1736–1743. 10.1212/WNL.0000000000002638 27037234 PMC4854589

[B36] Dickson D. W. (1999). Neuropathologic differentiation of progressive supranuclear palsy and corticobasal degeneration. J. Neurol. 246 (2), II6–II15. 10.1007/BF03161076 10525997

[B37] Donaghy P. C. McKeith I. G. (2014). The clinical characteristics of dementia with Lewy bodies and a consideration of prodromal diagnosis. Alzheimers Res. Ther. 6 (4), 46. 10.1186/alzrt274 25484925 PMC4255387

[B38] Drechsel D. N. Hyman A. A. Cobb M. H. Kirschner M. W. (1992). Modulation of the dynamic instability of tubulin assembly by the microtubule-associated protein tau. Mol. Biol. Cell 3 (10), 1141–1154. 10.1091/mbc.3.10.1141 1421571 PMC275678

[B39] Du Y. Wu H. T. Qin X. Y. Cao C. Liu Y. Cao Z. Z. (2018). Postmortem brain, cerebrospinal fluid, and blood neurotrophic factor levels in Alzheimer’s disease: a systematic review and meta-Analysis. J. Mol. Neurosci. 65 (3), 289–300. 10.1007/s12031-018-1100-8 29956088

[B40] Duka T. Duka V. Joyce J. N. Sidhu A. (2009). α-Synuclein contributes to GSK-3β-catalyzed tau phosphorylation in Parkinson’s disease models. FASEB J. 23 (9), 2820–2830. 10.1096/fj.08-120410 19369384 PMC2796901

[B41] Duka T. Rusnak M. Drolet R. E. Duka V. Wersinger C. Goudreau J. L. (2006). Alpha-synuclein induces hyperphosphorylation of tau in the MPTP model of parkinsonism. FASEB J. 20 (13), 2302–2312. 10.1096/fj.06-6092com 17077307

[B42] Fernandez-Botran R. Ahmed Z. Crespo F. A. Gatenbee C. Gonzalez J. Dickson D. W. (2011). Cytokine expression and microglial activation in progressive supranuclear palsy. Parkinsonism Relat. Disord. 17 (9), 683–688. 10.1016/j.parkreldis.2011.06.007 21741294 PMC3196843

[B43] Fernandez J. A. Rojo L. Kuljis R. O. Maccioni R. B. (2008). The damage signals hypothesis of Alzheimer’s disease pathogenesis. J. Alzheimers Dis. 14 (3), 329–333. 10.3233/JAD-2008-14307 18599959

[B44] Forno L. S. (1996). Neuropathology of Parkinson’s disease. J. Neuropathol. Exp. Neurol. 55 (3), 259–272. 10.1097/00005072-199603000-00001 8786384

[B45] Frankola K. A. Greig N.H. Luo W. Tweedie D. (2011). Targeting TNF-alpha to elucidate and ameliorate neuroinflammation in neurodegenerative diseases. CNS Neurol. Disord. Drug Targets 10 (3), 391–403. 10.2174/187152711794653751 21288189 PMC4663975

[B46] Frasier M. Walzer M. McCarthy L. Magnuson D. Lee J. M. Haas C. (2005). Tau phosphorylation increases in symptomatic mice overexpressing A30P α-synuclein. Exp. Neurol. 192 (2), 274–287. 10.1016/j.expneurol.2004.07.016 15755545

[B47] Friedman B. A. Srinivasan K. Ayalon G. Meilandt W. J. Lin H. Huntley M. A. (2018). Diverse brain myeloid expression profiles reveal distinct microglial activation states and aspects of Alzheimer’s disease not evident in mouse models. Cell Rep. 22 (3), 832–847. 10.1016/j.celrep.2017.12.066 29346778

[B48] Fulga T. A. Elson-Schwab I. Khurana V. Steinhilb M. L. Spires T. L. Hyman B. T. (2007). Abnormal bundling and accumulation of F-actin mediates tau-induced neuronal degeneration *in vivo*. Nat. Cell Biol. 9 (2), 139–148. 10.1038/ncb1528 17187063

[B49] Gagnon J. F. Bedard M. A. Fantini M. L. Petit D. Panisset M. Rompre S. (2002). REM sleep behavior disorder and REM sleep without atonia in Parkinson’s disease. Neurology 59 (4), 585–589. 10.1212/WNL.59.4.585 12196654

[B50] Garcia-Reitboeck P. Anichtchik O. Dalley J. W. Ninkina N. Tofaris G. K. Buchman V. L. (2013). Endogenous alpha-synuclein influences the number of dopaminergic neurons in mouse substantia nigra. Exp. Neurol. 248, 541–545. 10.1016/j.expneurol.2013.07.015 23933574 PMC4104299

[B51] Geekiyanage H. Chan C. (2011). MicroRNA-137/181c regulates serine palmitoyltransferase and in turn amyloid beta, novel targets in sporadic Alzheimer’s disease. J. Neurosci. 31 (41), 14820–14830. 10.1523/JNEUROSCI.3883-11.2011 21994399 PMC3200297

[B52] Ghetti B. Oblak A. L. Boeve B. F. Johnson K. A. Dickerson B. C. Goedert M. (2015). Invited review: frontotemporal dementia caused by microtubule-associated protein tau gene (MAPT) mutations: a chameleon for neuropathology and neuroimaging. Neuropathol. Appl. Neurobiol. 41 (1), 24–46. 10.1111/nan.12213 25556536 PMC4329416

[B53] Giannakopoulos P. Herrmann F. R. Bussiere T. Bouras C. Kovari E. Perl D. P. (2003). Tangle and neuron numbers, but not amyloid load, predict cognitive status in Alzheimer’s disease. Neurology 60 (9), 1495–1500. 10.1212/01.WNL.0000063311.58879.01 12743238

[B54] Giasson B. I. Forman M. S. Higuchi M. Golbe L. I. Graves C. L. Kotzbauer P. T. (2003). Initiation and synergistic fibrillization of tau and alpha-synuclein. Science 300 (5619), 636–640. 10.1126/science.1082324 12714745

[B55] Gibb W. R. Luthert P. J. Marsden C. D. (1989). Corticobasal degeneration. Brain 112 ( Pt 5), 1171–1192. 10.1093/brain/112.5.1171 2478251

[B56] Goedert M. Jakes R. Spillantini M. G. Hasegawa M. Smith M. J. Crowther R. A. (1996). Assembly of microtubule-associated protein tau into Alzheimer-like filaments induced by sulphated glycosaminoglycans. Nature 383 (6600), 550–553. 10.1038/383550a0 8849730

[B57] Goedert M. Spillantini M. G. Del Tredici K. Braak H. (2013). 100 years of Lewy pathology. Nat. Rev. Neurol. 9 (1), 13–24. 10.1038/nrneurol.2012.242 23183883

[B58] Golde T. E. Miller V. M. (2009). Proteinopathy-induced neuronal senescence: a hypothesis for brain failure in Alzheimer’s and other neurodegenerative diseases. Alzheimers Res. Ther. 1 (2), 5. 10.1186/alzrt5 19822029 PMC2874257

[B59] Grant R. E. Schneider J. A. Ferguson E. J. Cummings P. B. (1997). Total hip reconstruction in a woman with Cornelia de Lange syndrome: a case report. J. Natl. Med. Assoc. 89 (8), 530–532. 9264220 PMC2568114

[B60] Griciuc A. Serrano-Pozo A. Parrado A. R. Lesinski A. N. Asselin C. N. Mullin K. (2013). Alzheimer’s disease risk gene CD33 inhibits microglial uptake of amyloid beta. Neuron 78 (4), 631–643. 10.1016/j.neuron.2013.04.014 23623698 PMC3706457

[B61] Guillot-Sestier M. V. Doty K. R. Town T. (2015). Innate immunity fights Alzheimer’s disease. Trends Neurosci. 38 (11), 674–681. 10.1016/j.tins.2015.08.008 26549882 PMC4641041

[B62] Guzman-Martinez L. Farias G. A. Maccioni R. B. (2013). Tau oligomers as potential targets for Alzheimer’s diagnosis and novel drugs. Front. Neurol. 4, 167. 10.3389/fneur.2013.00167 24191153 PMC3808896

[B63] Haggerty T. Credle J. Rodriguez O. Wills J. Oaks A. W. Masliah E. (2011). Hyperphosphorylated tau in an α-synuclein-overexpressing transgenic model of Parkinson’s disease. Eur. J. Neurosci. 33 (9), 1598–1610. 10.1111/j.1460-9568.2011.07660.x 21453448 PMC3086951

[B64] Halliday G. M. Davies L. McRitchie D. A. Cartwright H. Pamphlett R. Morris J. G. L. (1995). Ubiquitin-positive achromatic neurons in corticobasal degeneration. Acta Neuropathol. 90 (1), 68–75. 10.1007/BF00294461 7572081

[B65] Harper D. G. Stopa E. G. Kuo-Leblanc V. McKee A. C. Asayama K. Volicer L. (2008). Dorsomedial SCN neuronal subpopulations subserve different functions in human dementia. Brain 131 (Pt 6), 1609–1617. 10.1093/brain/awn049 18372313 PMC3286014

[B66] Hebert S. S. Horre K. Nicolai L. Papadopoulou A. S. Mandemakers W. Silahtaroglu A. N. (2008). Loss of microRNA cluster miR-29a/b-1 in sporadic Alzheimer’s disease correlates with increased BACE1/beta-secretase expression. Proc. Natl. Acad. Sci. U.S.A. 105 (17), 6415–6420. 10.1073/pnas.0710263105 18434550 PMC2359789

[B67] Heneka M. T. Golenbock D. T. Latz E. (2015). Innate immunity in Alzheimer’s disease. Nat. Immunol. 16 (3), 229–236. 10.1038/ni.3102 25689443

[B68] Hodges J. R. Davies R. Xuereb J. Kril J. Halliday G. (2003). Survival in frontotemporal dementia. Neurology 61 (3), 349–354. 10.1212/01.WNL.0000078928.20107.52 12913196

[B69] Hol E. M. Pekny M. (2015). Glial fibrillary acidic protein (GFAP) and the astrocyte intermediate filament system in diseases of the central nervous system. Curr. Opin. Cell Biol. 32, 121–130. 10.1016/j.ceb.2015.02.004 25726916

[B70] Holtzman D. M. Bales K. R. Tenkova T. Fagan A. M. Parsadanian M. Sartorius L. J. (2000). Apolipoprotein E isoform-dependent amyloid deposition and neuritic degeneration in a mouse model of Alzheimer’s disease. Proc. Natl. Acad. Sci. 97 (6), 2892–2897. 10.1073/pnas.050004797 10694577 PMC16026

[B71] Hughes L. E. Rittman T. Regenthal R. Robbins T. W. Rowe J. B. (2015). Improving response inhibition systems in frontotemporal dementia with citalopram. Brain 138 (Pt 7), 1961–1975. 10.1093/brain/awv133 26001387 PMC5412666

[B72] Irwin D. J. Cairns N. J. Grossman M. McMillan C. T. Lee E. B. Van Deerlin V. M. (2015). Frontotemporal lobar degeneration: defining phenotypic diversity through personalized medicine. Acta Neuropathol. 129 (4), 469–491. 10.1007/s00401-014-1380-1 25549971 PMC4369168

[B73] Iseki E. Togo T. Suzuki K. Katsuse O. Marui W. de Silva R. (2003). Dementia with Lewy bodies from the perspective of tauopathy. Acta Neuropathol. 105 (3), 265–270. 10.1007/s00401-002-0644-3 12557014

[B74] Jacobs A. H. Tavitian B. (2012). Noninvasive molecular imaging of neuroinflammation. J. Cereb. Blood Flow Metab. 32 (7), 1393–1415. 10.1038/jcbfm.2012.53 22549622 PMC3390799

[B75] Jellinger K. A. (2010a). Basic mechanisms of neurodegeneration: a critical update. J. Cell. Mol. Med. 14 (3), 457–487. 10.1111/j.1582-4934.2010.01010.x 20070435 PMC3823450

[B76] Jellinger K. A. (2010b). The neuropathologic substrate of Parkinson disease dementia. Acta Neuropathol. 119 (1), 151–153. 10.1007/s00401-009-0613-1 19924423

[B77] Jellinger K. A. (2011). Interaction between α-synuclein and tau in Parkinson’s disease: comment on Wills et al.: elevated tauopathy and α-synuclein pathology in postmortem Parkinson’s disease brains with and without dementia. Exp Neurol 2010; 225: 210-218. Exp. Neurol. 227 (1), 13–18. 10.1016/j.expneurol.2010.10.006 20965169

[B78] Jensen P. H. Hager H. Nielsen M. S. Hojrup P. Gliemann J. Jakes R. (1999). α-Synuclein binds to tau and stimulates the protein kinase A-catalyzed tau phosphorylation of serine residues 262 and 356. J. Biol. Chem. 274 (36), 25481–25489. 10.1074/jbc.274.36.25481 10464279

[B79] Jha M. K. Jo M. Kim J. H. Suk K. (2019). Microglia–astrocyte crosstalk: an intimate molecular conversation. Neuroscientist 25 (3), 227–240. 10.1177/1073858418783959 29931997

[B80] Joachim C. L. Morris J. H. Kosik K. S. Selkoe D. J. (1987). Tau antisera recognize neurofibrillary tangles in a range of neurodegenerative disorders. Ann. Neurol. 22 (4), 514–520. 10.1002/ana.410220411 2963585

[B81] Jochum W. Hanggi D. Bruder E. Jeck T. Novotny H. Probst A. (2004). Inflammatory myofibroblastic tumour of the sella turcica. Neuropathol. Appl. Neurobiol. 30 (6), 692–695. 10.1111/j.1365-2990.2004.00611.x 15541009

[B82] Jonsson T. Stefansson H. Steinberg S. Jonsdottir I. Jonsson P. V. Snaedal J. (2013). Variant of TREM2 associated with the risk of Alzheimer’s disease. N. Engl. J. Med. 368 (2), 107–116. 10.1056/NEJMoa1211103 23150908 PMC3677583

[B83] Josephs K. A. (2010). “Corticobasal ganglionic degeneration,” in Blue books of neurology. Eds. Schapira A. H. V. Lang A. E. T. Fahn S. (Butterworth-Heinemann), 375–396. 10.1016/B978-1-4160-6641-5.00022-2

[B84] Josephs K. A. Petersen R. C. Knopman D. S. Boeve B. F. Whitwell J. L. Duffy J. R. (2006a). Clinicopathologic analysis of frontotemporal and corticobasal degenerations and PSP. Neurology 66 (1), 41–48. 10.1212/01.wnl.0000191307.69661.c3 16401843

[B85] Josephs K. A. Whitwell J. L. Boeve B. F. Knopman D. S. Tang-Wai D. F. Drubach D. A. (2006b). Visual hallucinations in posterior cortical atrophy. Arch. Neurol. 63 (10), 1427–1432. 10.1001/archneur.63.10.1427 17030659 PMC2748870

[B86] Kandimalla R. J. Anand R. Veeramanikandan R. Wani W. Y. Prabhakar S. Grover V. K. (2014). CSF ubiquitin as a specific biomarker in Alzheimer’s disease. Curr. Alzheimer Res. 11 (4), 340–348. 10.2174/1567205011666140331161027 24720893

[B87] Kandimalla R. J. Prabhakar S. Binukumar B. K. Wani W. Y. Gupta N. Sharma D. R. (2011a). Apo-epsilon4 allele in conjunction with Abeta42 and tau in CSF: biomarker for Alzheimer’s disease. Curr. Alzheimer Res. 8 (2), 187–196. 10.2174/156720511795256071 21222606

[B88] Kandimalla R. J. Prabhakar S. Binukumar B. K. Wani W. Y. Sharma D. R. Grover V. K. (2011b). Cerebrospinal fluid profile of amyloid beta42 (Abeta42), hTau and ubiquitin in North Indian Alzheimer’s disease patients. Neurosci Lett 487 (2), 134–138. 10.1016/j.neulet.2010.06.075 20599474

[B89] Kandimalla R. J. Prabhakar S. Wani W. Y. Kaushal A. Gupta N. Sharma D. R. (2013). CSF p-tau levels in the prediction of Alzheimer’s disease. Biol. Open 2 (11), 1119–1124. 10.1242/bio.20135447 24244848 PMC3828758

[B90] Kaul T. Credle J. Haggerty T. Oaks A. W. Masliah E. Sidhu A. (2011). Region-specific tauopathy and synucleinopathy in brain of the alpha-synuclein overexpressing mouse model of Parkinson’s disease. BMC Neurosci. 12, 79. 10.1186/1471-2202-12-79 21812967 PMC3176190

[B91] Kawakami F. Suzuki M. Shimada N. Kagiya G. Ohta E. Tamura K. (2011). Stimulatory effect of alpha-synuclein on the tau-phosphorylation by GSK-3beta. FEBS J. 278 (24), 4895–4904. 10.1111/j.1742-4658.2011.08389.x 21985244

[B92] Kempster P. A. Williams D. R. Selikhova M. Holton J. Revesz T. Lees A. J. (2007). Patterns of levodopa response in Parkinson’s disease: a clinico-pathological study. Brain 130 (Pt 8), 2123–2128. 10.1093/brain/awm142 17586867

[B93] Keren-Shaul H. Spinrad A. Weiner A. Matcovitch-Natan O. Dvir-Szternfeld R. Ulland T. K. (2017). A unique microglia type associated with restricting development of Alzheimer’s disease. Cell 169 (7), 1276–1290, e1217. 10.1016/j.cell.2017.05.018 28602351

[B94] King A. Al-Sarraj S. Troakes C. Smith B. N. Maekawa S. Iovino M. (2013). Mixed tau, TDP-43 and p62 pathology in FTLD associated with a C9ORF72 repeat expansion and p.Ala239Thr MAPT (tau) variant. Acta Neuropathol. 125 (2), 303–310. 10.1007/s00401-012-1050-0 23053136

[B95] Knopman D. S. Roberts R. O. (2011). Estimating the number of persons with frontotemporal lobar degeneration in the US population. J. Mol. Neurosci. 45 (3), 330–335. 10.1007/s12031-011-9538-y 21584654 PMC3208074

[B96] Koenigsknecht-Talboo J. Landreth G. E. (2005). Microglial phagocytosis induced by fibrillar beta-amyloid and IgGs are differentially regulated by proinflammatory cytokines. J. Neurosci. 25 (36), 8240–8249. 10.1523/JNEUROSCI.1808-05.2005 16148231 PMC6725530

[B97] Koistinaho M. Lin S. Wu X. Esterman M. Koger D. Hanson J. (2004). Apolipoprotein E promotes astrocyte colocalization and degradation of deposited amyloid-β peptides. Nat. Med. 10 (7), 719. 10.1038/nm1058 15195085

[B98] Kovacs G. G. (2017). Tauopathies. Handb. Clin. Neurol. 145, 355–368. 10.1016/B978-0-12-802395-2.00025-0 28987182

[B99] Kovacs G. G. Botond G. Budka H. (2010). Protein coding of neurodegenerative dementias: the neuropathological basis of biomarker diagnostics. Acta Neuropathol. 119 (4), 389–408. 10.1007/s00401-010-0658-1 20198481

[B100] Kovari E. Gold G. Herrmann F. R. Canuto A. Hof P. R. Bouras C. (2003). Lewy body densities in the entorhinal and anterior cingulate cortex predict cognitive deficits in Parkinson’s disease. Acta Neuropathol. 106 (1), 83–88. 10.1007/s00401-003-0705-2 12687392

[B101] Kraybill M. L. Larson E. B. Tsuang D. W. Teri L. McCormick W. C. Bowen J. D. (2005). Cognitive differences in dementia patients with autopsy-verified AD, Lewy body pathology, or both. Neurology 64 (12), 2069–2073. 10.1212/01.WNL.0000165987.89198.65 15985574 PMC1513627

[B102] Krishnamurthy P. K. Deng Y. Sigurdsson E. M. (2011). Mechanistic studies of antibody-mediated clearance of tau aggregates using an *ex vivo* brain slice model. Front. Psychiatry 21, 2:59. 10.3389/fpsyt.2011.00059.eCollection2011. PMC3198029 22025915

[B103] Laihinen A. O. Rinne J. O. Ruottinen H. M. Nagren K. A. Lehikoinen P. K. Oikonen V. J. (1994). PET studies on dopamine D1 receptors in the human brain with carbon-11-SCH 39166 and carbon-11-NNC 756. J. Nucl. Med. 35 (12), 1916–1920. 7989969

[B104] Langlais P. J. Thal L. Hansen L. Galasko D. Alford M. Masliah E. (1993). Neurotransmitters in basal ganglia and cortex of Alzheimer’s disease with and without Lewy bodies. Neurology 43 (10), 1927–1934. 10.1212/WNL.43.10.1927 8105420

[B105] Lee V. M. Giasson B. I. Trojanowski J. Q. (2004). More than just two peas in a pod: common amyloidogenic properties of tau and alpha-synuclein in neurodegenerative diseases. Trends Neurosci. 27 (3), 129–134. 10.1016/j.tins.2004.01.007 15036877

[B106] Lee M. S. Rinne J. O. Ceballos-Baumann A. Thompson P. D. Marsden C. D. (1994). Dystonia after head trauma. Neurology 44 (8), 1374–1378. 10.1212/WNL.44.8.1374 8058132

[B107] Leidinger P. Backes C. Deutscher S. Schmitt K. Mueller S. C. Frese K. (2013). A blood based 12-miRNA signature of Alzheimer disease patients. Genome Biol. 14 (7), R78. 10.1186/gb-2013-14-7-r78 23895045 PMC4053778

[B108] Leyns C. E. G. Holtzman D. M. (2017). Glial contributions to neurodegeneration in tauopathies. Mol. Neurodegener. 12 (1), 50. 10.1186/s13024-017-0192-x 28662669 PMC5492997

[B109] Lian H. Yang L. Cole A. Sun L. Chiang A. C.-A. Fowler S. W. (2015). NFκB-activated astroglial release of complement C3 compromises neuronal morphology and function associated with Alzheimer’s disease. Neuron 85 (1), 101–115. 10.1016/j.neuron.2014.11.018 25533482 PMC4289109

[B110] Ling H. (2016). Clinical approach to progressive supranuclear palsy. JMD 9 (1), 3–13. 10.14802/jmd.15060 26828211 PMC4734991

[B111] Lopez Gonzalez I. Garcia-Esparcia P. Llorens F. Ferrer I. (2016). Genetic and transcriptomic profiles of inflammation in neurodegenerative diseases: Alzheimer, Parkinson, Creutzfeldt-Jakob and tauopathies. Int. J. Mol. Sci. 17 (2), 206. 10.3390/ijms17020206 26861289 PMC4783939

[B112] Lull M. E. Block M. L. (2010). Microglial activation and chronic neurodegeneration. Neurotherapeutics 7 (4), 354–365. 10.1016/j.nurt.2010.05.014 20880500 PMC2951017

[B113] Maarouf C. L. Beach T. G. Adler C. H. Shill H. A. Sabbagh M. N. Wu T. (2012). Cerebrospinal fluid biomarkers of neuropathologically diagnosed Parkinson’s disease subjects. Neurol. Res. 34 (7), 669–676. 10.1179/1743132812Y.0000000063 22889670 PMC3681206

[B114] Maccioni R. B. (2011). Tau protein in Alzheimer’s disease. Curr. Alzheimer Res. 8 (6), 607. 10.2174/156720511796717159 21827396

[B115] Maccioni R. B. (2012). Introductory remarks. Molecular, biological and clinical aspects of Alzheimer’s disease. Arch. Med. Res. 43 (8), 593–594. 10.1016/j.arcmed.2012.11.001 23149157

[B116] Maccioni R. B. Rojo L. E. Fernandez J. A. Kuljis R. O. (2009). The role of neuroimmunomodulation in Alzheimer’s disease. Ann. N. Y. Acad. Sci. 1153, 240–246. 10.1111/j.1749-6632.2008.03972.x 19236346

[B117] Mackenzie I. R. Neumann M. Bigio E. H. Cairns N. J. Alafuzoff I. Kril J. (2010). Nomenclature and nosology for neuropathologic subtypes of frontotemporal lobar degeneration: an update. Acta Neuropathol. 119 (1), 1–4. 10.1007/s00401-009-0612-2 19924424 PMC2799633

[B118] Maeda J. Zhang M. R. Okauchi T. Ji B. Ono M. Hattori S. (2011). *In vivo* positron emission tomographic imaging of glial responses to amyloid-beta and tau pathologies in mouse models of Alzheimer’s disease and related disorders. J. Neurosci. 31 (12), 4720–4730. 10.1523/JNEUROSCI.3076-10.2011 21430171 PMC3251921

[B119] Mahapatra R. K. Edwards M. J. Schott J. M. Bhatia K. P. (2004). Corticobasal degeneration. Lancet Neurol. 3 (12), 736–743. 10.1016/S1474-4422(04)00936-6 15556806

[B120] Maldonado-Lasuncion I. Atienza M. Sanchez-Espinosa M. P. Cantero J. L. (2018). Aging-related changes in cognition and cortical integrity are associated with serum expression of candidate microRNAs for Alzheimer disease. Cereb. Cortex. 2018, 1–12. 10.1093/cercor/bhy323 30590432

[B121] Malia T. J. Teplyakov A. Ernst R. Wu S. J. Lacy E. R. Liu X. (2016). Epitope mapping and structural basis for the recognition of phosphorylated tau by the anti-tau antibody AT8. Proteins 84 (4), 427–434. 10.1002/prot.24988 26800003 PMC5067699

[B122] Maphis N. Xu G. Kokiko-Cochran O. N. Jiang S. Cardona A. Ransohoff R. M. (2015). Reactive microglia drive tau pathology and contribute to the spreading of pathological tau in the brain. Brain 138 (6), 1738–1755. 10.1093/brain/awv081 25833819 PMC4542622

[B123] Martin H. L. Santoro M. Mustafa S. Riedel G. Forrester J. V. Teismann P. (2016). Evidence for a role of adaptive immune response in the disease pathogenesis of the MPTP mouse model of Parkinson’s disease. Glia 64 (3), 386–395. 10.1002/glia.22935 26511587 PMC4855685

[B124] Martinez B. Peplow P. V. (2019). MicroRNAs as diagnostic and therapeutic tools for Alzheimer’s disease: advances and limitations. Neural Regen. Res. 14 (2), 242–255. 10.4103/1673-5374.244784 30531004 PMC6301178

[B125] Matsumoto S. Udaka F. Kameyama M. Kusaka H. Ito H. Imai T. (1996). Subcortical neurofibrillary tangles, neuropil threads, and argentophilic glial inclusions in corticobasal degeneration. Clin. Neuropathol. 15 (4), 209–214. 8836605

[B126] McKee A. C. Carreras I. Hossain L. Ryu H. Klein W. L. Oddo S. (2008). Ibuprofen reduces Abeta, hyperphosphorylated tau and memory deficits in Alzheimer mice. Brain Res. 1207, 225–236. 10.1016/j.brainres.2008.01.095 18374906 PMC2587244

[B127] McKeith I. G. Dickson D. W. Lowe J. Emre M. O’Brien J. T. Feldman H. (2005). Diagnosis and management of dementia with Lewy bodies: third report of the DLB Consortium. Neurology 65 (12), 1863–1872. 10.1212/01.wnl.0000187889.17253.b1 16237129

[B128] Meda L. Baron P. Scarlato G. (2001). Glial activation in Alzheimer’s disease: the role of Abeta and its associated proteins. Neurobiol. Aging 22 (6), 885–893. 10.1016/S0197-4580(01)00307-4 11754995

[B129] Medeiros R. LaFerla F. M. (2013). Astrocytes: conductors of the Alzheimer disease neuroinflammatory symphony. Exp. Neurol. 239, 133–138. 10.1016/j.expneurol.2012.10.007 23063604

[B130] Mirra S. S. Murrell J. R. Gearing M. Spillantini M. G. Goedert M. Crowther R. A. (1999). Tau pathology in a family with dementia and a P301L mutation in tau. J. Neuropathol. Exp. Neurol. 58 (4), 335–345. 10.1097/00005072-199904000-00004 10218629

[B131] Morales I. Farias G. Maccioni R. B. (2010). Neuroimmunomodulation in the pathogenesis of Alzheimer’s disease. Neuroimmunomodulation 17 (3), 202–204. 10.1159/000258724 20134203

[B132] Morales I. Guzman-Martinez L. Cerda-Troncoso C. Farias G. A. Maccioni R. B. (2014). Neuroinflammation in the pathogenesis of Alzheimer’s disease. A rational framework for the search of novel therapeutic approaches. Front. Cell. Neurosci. 8, 112. 10.3389/fncel.2014.00112 24795567 PMC4001039

[B133] Morales I. Jimenez J. M. Mancilla M. Maccioni R. B. (2013). Tau oligomers and fibrils induce activation of microglial cells. J. Alzheimers Dis. 37 (4), 849–856. 10.3233/JAD-131843 23948931

[B134] Mulder S. D. Veerhuis R. Blankenstein M. A. Nielsen H. M. (2012). The effect of amyloid associated proteins on the expression of genes involved in amyloid-β clearance by adult human astrocytes. Exp. Neurol. 233 (1), 373–379. 10.1016/j.expneurol.2011.11.001 22101005

[B135] Muntane G. Dalfó E. Martinez A. Ferrer I. (2008). Phosphorylation of tau and alpha-synuclein in synaptic-enriched fractions of the frontal cortex in Alzheimer's disease, and in Parkinson's disease and related alpha-synucleinopathies. Neuroscience 152 (4), 913–923. 10.1016/j.neuroscience.2008.01.030 18343584

[B136] Musiek E. S. Holtzman D. M. (2015). Three dimensions of the amyloid hypothesis: time, space and ‘wingmen’. Nat. Neurosci. 18 (6), 800–806. 10.1038/nn.4018 26007213 PMC4445458

[B137] Nagaraj S. Laskowska-Kaszub K. Debski K. J. Wojsiat J. Dabrowski M. Gabryelewicz T. (2017). Profile of 6 microRNA in blood plasma distinguish early stage Alzheimer’s disease patients from non-demented subjects. Oncotarget 8 (10), 16122–16143. 10.18632/oncotarget.15109 28179587 PMC5369952

[B138] Neary D. Snowden J. S. Gustafson L. Passant U. Stuss D. Black S. (1998). Frontotemporal lobar degeneration: a consensus on clinical diagnostic criteria. Neurology 51 (6), 1546–1554. 10.1212/WNL.51.6.1546 9855500

[B139] Neary D. Snowden J. S. Northen B. Goulding P. (1988). Dementia of frontal-lobe type. J. Neurol. Neurosurg. Psychiatry 51 (3), 353–361. 10.1136/jnnp.51.3.353 3258902 PMC1032860

[B140] Neumann K. Farias G. Slachevsky A. Perez P. Maccioni R. B. (2011). Human platelets tau: a potential peripheral marker for Alzheimer’s disease. J. Alzheimers Dis. 25 (1), 103–109. 10.3233/JAD-2011-101641 21368381

[B141] Nimmerjahn A. Kirchhoff F. Helmchen F. (2005). Resting microglial cells are highly dynamic surveillants of brain parenchyma *in vivo*. Science 308 (5726), 1314–1318. 10.1126/science.1110647 15831717

[B142] Nubling G. Bader B. Levin J. Hildebrandt J. Kretzschmar H. Giese A. (2012). Synergistic influence of phosphorylation and metal ions on tau oligomer formation and coaggregation with alpha-synuclein at the single molecule level. Mol. Neurodegener. 7, 35. 10.1186/1750-1326-7-35 22824345 PMC3472288

[B143] Oaks A. W. Frankfurt M. Finkelstein D. I. Sidhu A. (2013). Age-dependent effects of A53T alpha-synuclein on behavior and dopaminergic function. PLoS One 8 (4), e60378. 10.1371/journal.pone.0060378 23560093 PMC3613356

[B144] Olichney J. M. Galasko D. Salmon D. P. Hofstetter C. R. Hansen L. A. Katzman R. (1998). Cognitive decline is faster in Lewy body variant than in Alzheimer’s disease. Neurology 51 (2), 351–357. 10.1212/WNL.51.2.351 9710002

[B145] Olney N. T. Spina S. Miller B. L. (2017). Frontotemporal dementia. Neurol. Clin. 35 (2), 339–374. 10.1016/j.ncl.2017.01.008 28410663 PMC5472209

[B146] Onyike C. U. Diehl-Schmid J. (2013). The epidemiology of frontotemporal dementia. Int. Rev. Psychiatry 25 (2), 130–137. 10.3109/09540261.2013.776523 23611343 PMC3932112

[B147] Orr M. E. Sullivan A. C. Frost B. (2017). A brief overview of tauopathy: causes, consequences, and therapeutic strategies. Trends Pharmacol. Sci. 38, 637–648. . 101016/j.tips.201703.011 28455089 10.1016/j.tips.2017.03.011 PMC5476494

[B148] Pal R. Tiwari P. C. Nath R. Pant K. K. (2016). Role of neuroinflammation and latent transcription factors in pathogenesis of Parkinson’s disease. Neurol. Res. 38 (12), 1111–1122. 10.1080/01616412.2016.1249997 27808010

[B149] Paolicelli R. C. Bisht K. Tremblay M. E. (2014). Fractalkine regulation of microglial physiology and consequences on the brain and behavior. Front. Cell. Neurosci. 8, 129. 10.3389/fncel.2014.00129 24860431 PMC4026677

[B150] Parhizkar S. Arzberger T. Brendel M. Kleinberger G. Deussing M. Focke C. (2019). Loss of TREM2 function increases amyloid seeding but reduces plaque-associated ApoE. Nat. Neurosci. 22, 191–204. 10.1038/s41593-018-0296-9 30617257 PMC6417433

[B151] Phillips E. C. Croft C. L. Kurbatskaya K. O’Neill M. J. Hutton M. L. Hanger D. P. (2014). Astrocytes and neuroinflammation in Alzheimer’s disease. Biochem. Soc. Trans. 42 (5), 1321–1325. 10.1042/BST20140155 25233410

[B152] Ransohoff R. M. (2016a). How neuroinflammation contributes to neurodegeneration. Science 353 (6301), 777–783. 10.1126/science.aag2590 27540165

[B153] Ransohoff R. M. (2016b). A polarizing question: do M1 and M2 microglia exist? Nat. Neurosci. 19 (8), 987. 10.1038/nn.4338 27459405

[B154] Rascovsky K. Hodges J. R. Knopman D. Mendez M. F. Kramer J. H. Neuhaus J. (2011). Sensitivity of revised diagnostic criteria for the behavioural variant of frontotemporal dementia. Brain 134 (Pt 9), 2456–2477. 10.1093/brain/awr179 21810890 PMC3170532

[B155] Rebeiz J. J. Kolodny E. H. Richardson E. P. Jr. (1968). Corticodentatonigral degeneration with neuronal achromasia. Arch. Neurol. 18 (1), 20–33. 10.1001/archneur.1968.00470310034003 5634369

[B156] Riedel M. Goldbaum O. Richter-Landsberg C. (2009). α-Synuclein promotes the recruitment of tau to protein inclusions in oligodendroglial cells: effects of oxidative and proteolytic stress. J. Mol. Neurosci. 39 (1-2), 226–234. 10.1007/s12031-009-9190-y 19266322

[B157] Riederer I. M. Pfulg C. Bouras C. Giannakopoulos P. Riederer B. M. (2003). Human immunoglobulins and Fc fragments promote microtubule assembly *via* tau proteins and induce conformational changes of neuronal microtubules *in vitro*. Neuroreport 14 (1), 117–121. 10.1097/00001756-200301200-00022 12544842

[B158] Rinne J. O. Daniel S. E. Scaravilli F. Harding A. E. Marsden C. D. (1994a). Nigral degeneration in neuroacanthocytosis. Neurology 44 (9), 1629–1632. 10.1212/WNL.44.9.1629 7936287

[B159] Rinne J. O. Daniel S. E. Scaravilli F. Pires M. Harding A. E. Marsden C. D. (1994b). The neuropathological features of neuroacanthocytosis. Mov. Disord. 9 (3), 297–304. 10.1002/mds.870090303 8041370

[B160] Rinne J. O. Lee M. S. Thompson P. D. Marsden C. D. (1994c). Corticobasal degeneration. A clinical study of 36 cases. Brain 117 ( Pt 5), 1183–1196. 10.1093/brain/117.5.1183 7953598

[B161] Rojo L. E. Fernandez J. A. Maccioni A. A. Jimenez J. M. Maccioni R. B. (2008). Neuroinflammation: implications for the pathogenesis and molecular diagnosis of Alzheimer’s disease. Arch. Med. Res. 39 (1), 1–16. 10.1016/j.arcmed.2007.10.001 18067990

[B162] Salmon E. Bernard Ir C. Hustinx R. (2015). Pitfalls and limitations of PET/CT in brain imaging. Semin. Nucl. Med. 45 (6), 541–551. 10.1053/j.semnuclmed.2015.03.008 26522395

[B163] Salonen R. Rinne J. O. Halonen P. Puusa A. Marttila R. Viljanen M. K. (1994). Lyme borreliosis associated with complete flaccid paraplegia. J. Infect. 28 (2), 181–184. 10.1016/S0163-4453(94)95660-X 8034998

[B164] Sarazin M. Michon A. Pillon B. Samson Y. Canuto A. Gold G. (2003). Metabolic correlates of behavioral and affective disturbances in frontal lobe pathologies. J. Neurol. 250 (7), 827–833. 10.1007/s00415-003-1087-z 12883925

[B165] Schmidt M. L. Zhukareva V. Perl D. P. Sheridan S. K. Schuck T. Lee V. M. (2001). Spinal cord neurofibrillary pathology in Alzheimer disease and Guam parkinsonism–dementia complex. J. Neuropathol. Exp. Neurol. 60 (11), 1075–1086. 10.1093/jnen/60.11.1075 11706937

[B166] Schneider J. A. Li J. L. Li Y. Wilson R. S. Kordower J. H. Bennett D. A. (2006). Substantia nigra tangles are related to gait impairment in older persons. Ann. Neurol. 59 (1), 166–173. 10.1002/ana.20723 16374822

[B167] Schneider J. A. Watts R. L. Gearing M. Brewer R. P. Mirra S. S. (1997). Corticobasal degeneration: neuropathologic and clinical heterogeneity. Neurology 48 (4), 959–969. 10.1212/WNL.48.4.959 9109885

[B168] Shao C. Y. Crary J. F. Rao C. Sacktor T. C. Mirra S. S. (2006). Atypical protein kinase C in neurodegenerative disease II: PKCiota/lambda in tauopathies and alpha-synucleinopathies. J. Neuropathol. Exp. Neurol. 65 (4), 327–335. 10.1097/01.jnen.0000218441.00040.82 16691114

[B169] Shastri A. Bonifati D. M. Kishore U. (2013). Innate immunity and neuroinflammation. Mediators Inflamm. 2013, 342931. 10.1155/2013/342931 23843682 PMC3697414

[B170] Sieben A. Van Langenhove T. Engelborghs S. Martin J. J. Boon P. Cras P. (2012). The genetics and neuropathology of frontotemporal lobar degeneration. Acta Neuropathol. 124 (3), 353–372. 10.1007/s00401-012-1029-x 22890575 PMC3422616

[B171] Sims R. Van Der Lee S. J. Naj A. C. Bellenguez C. Badarinarayan N. Jakobsdottir J. (2017). Rare coding variants in PLCG2, ABI3, and TREM2 implicate microglial-mediated innate immunity in Alzheimer’s disease. Nat. Genet. 49 (9), 1373. 10.1038/ng.3916 28714976 PMC5669039

[B172] Sjodin S. Hansson O. Ohrfelt A. Brinkmalm G. Zetterberg H. Brinkmalm A. (2017). Mass spectrometric analysis of cerebrospinal fluid ubiquitin in Alzheimer’s disease and Parkinsonian disorders. Proteomics Clin. Appl. 11, 1–9, 1700100. 10.1002/prca.201700100 PMC5765402 28972305

[B173] Sofroniew M. V. Vinters H. V. (2010). Astrocytes: biology and pathology. Acta Neuropathol. 119 (1), 7–35. 10.1007/s00401-009-0619-8 20012068 PMC2799634

[B174] Spillantini M. G. Goedert M. (2013). Tau pathology and neurodegeneration. Lancet Neurol. 12 (6), 609–622. 10.1016/S1474-4422(13)70090-5 23684085

[B175] Spillantini M. G. Yoshida H. Rizzini C. Lantos P. L. Khan N. Rossor M. N. (2000). A novel tau mutation (N296N) in familial dementia with swollen achromatic neurons and corticobasal inclusion bodies. Ann. Neurol. 48 (6), 939–943. 10.1002/1531-8249(200012)48:6<939::AID-ANA17>3.0.CO;2-1 11117553

[B176] Tang-Wai D. F. Josephs K. A. Boeve B. F. Dickson D. W. Parisi J. E. Petersen R. C. (2003a). Pathologically confirmed corticobasal degeneration presenting with visuospatial dysfunction. Neurology 61 (8), 1134–1135. 10.1212/01.WNL.0000086814.35352.B3 14581681

[B177] Tang-Wai D. F. Knopman D. S. Geda Y. E. Edland S. D. Smith G. E. Ivnik R. J. (2003b). Comparison of the short test of mental status and the mini-mental state examination in mild cognitive impairment. Arch. Neurol. 60 (12), 1777–1781. 10.1001/archneur.60.12.1777 14676056

[B178] Thomas B. Beal M. F. (2007). Parkinson’s disease. Hum. Mol. Genet. 16 Spec No2, R183–R194. 10.1093/hmg/ddm159 17911161

[B179] Tolnay M. Clavaguera F. (2004). Argyrophilic grain disease: a late-onset dementia with distinctive features among tauopathies. Neuropathology 24 (4), 269–283. 10.1111/j.1440-1789.2004.00591.x 15641585

[B180] Trojanowski J. Q. Dickson D. (2001). Update on the neuropathological diagnosis of frontotemporal dementias. J. Neuropathol. Exp. Neurol. 60 (12), 1123–1126. 10.1093/jnen/60.12.1123 11764085

[B181] Tsuchiya K. Ikeda K. Uchihara T. Oda T. Shimada H. (1997). Distribution of cerebral cortical lesions in corticobasal degeneration: a clinicopathological study of five autopsy cases in Japan. Acta Neuropathol. 94 (5), 416–424. 10.1007/s004010050728 9386773

[B182] Uchihara T. Nakayama H. (2006). Familial tauopathy mimicking corticobasal degeneration an autopsy study on three siblings. J. Neurol. Sci. 246 (1-2), 45–51. 10.1016/j.jns.2006.02.005 16540122

[B183] Uversky V. N. (2007). Neuropathology, biochemistry, and biophysics of alpha-synuclein aggregation. J. Neurochem. 103 (1), 17–37. 10.1111/j.1471-4159.2007.04764.x 17623039

[B184] Uversky V. N. (2009). Intrinsically disordered proteins and their environment: effects of strong denaturants, temperature, pH, counter ions, membranes, binding partners, osmolytes, and macromolecular crowding. Protein J. 28 (7-8), 305–325. 10.1007/s10930-009-9201-4 19768526

[B185] Venneti S. Wiley C. A. Kofler J. (2009). Imaging microglial activation during neuroinflammation and Alzheimer’s disease. J. Neuroimmune Pharmacol. 4 (2), 227–243. 10.1007/s11481-008-9142-2 19052878 PMC2682630

[B186] Violet M. Delattre L. Tardivel M. Sultan A. Chauderlier A. Caillierez R. (2014). A major role for tau in neuronal DNA and RNA protection *in vivo* under physiological and hyperthermic conditions. Front. Cell. Neurosci. 8, 84. 10.3389/fncel.2014.00084 24672431 PMC3957276

[B187] Walter S. Letiembre M. Liu Y. Heine H. Penke B. Hao W. (2007). Role of the toll-like receptor 4 in neuroinflammation in Alzheimer’s disease. Cell. Physiol. Biochem. 20 (6), 947–956. 10.1159/000110455 17982277

[B188] Wang Y. Mandelkow E. (2015). Tau in physiology and pathology. Nat. Rev. Neurosci. 17 (1), 5–21. 10.1038/nrn.2015.1 26631930

[B189] Wang L. Jiang Q. Chu J. Lin L. Li X. G. Chai G. S. (2013). Expression of Tau40 induces activation of cultured rat microglial cells. PLoS One 8 (10), e76057. 10.1371/journal.pone.0076057 24146816 PMC3795725

[B190] Wang W. Y. Tan M. S. Yu J. T. Tan L. (2015). Role of pro-inflammatory cytokines released from microglia in Alzheimer’s disease. Ann. Transl. Med. 3 (10), 136. 10.3978/j.issn.2305-5839.2015.03.49 26207229 PMC4486922

[B191] Wani W. Y. Gudup S. Sunkaria A. Bal A. Singh P. P. Kandimalla R. J. (2011). Protective efficacy of mitochondrial targeted antioxidant MitoQ against dichlorvos induced oxidative stress and cell death in rat brain. Neuropharmacology 61 (8), 1193–1201. 10.1016/j.neuropharm.2011.07.008 21784090

[B192] Wani W. Y. Sunkaria A. Sharma D. R. Kandimalla R. J. Kaushal A. Gerace E. (2014). Caspase inhibition augments dichlorvos-induced dopaminergic neuronal cell death by increasing ROS production and PARP1 activation. Neuroscience 258, 1–15. 10.1016/j.neuroscience.2013.11.004 24231740

[B193] Wei H. Zhu X. Li Y. (2018). Application value of serum biomarkers for choosing memantine therapy for moderate AD. J. Neurol. 265 (8), 1844–1849. 10.1007/s00415-018-8926-4 29948244

[B194] Weingarten M. D. Lockwood A. H. Hwo S. Y. Kirschner M. W. (1975). A protein factor essential for microtubule assembly. Proc. Natl. Acad. Sci. U.S.A. 72 (5), 1858–1862. 10.1073/pnas.72.5.1858 1057175 PMC432646

[B195] Wenning G. K. Litvan I. Jankovic J. Granata R. Mangone C. A. McKee A. (1998). Natural history and survival of 14 patients with corticobasal degeneration confirmed at postmortem examination. J. Neurol. Neurosurg. Psychiatry 64 (2), 184–189. 10.1136/jnnp.64.2.184 9489528 PMC2169933

[B196] Wes P. D. Easton A. Corradi J. Barten D. M. Devidze N. DeCarr L. B. (2014). Tau overexpression impacts a neuroinflammation gene expression network perturbed in Alzheimer’s disease. PLoS One 9 (8), e106050. 10.1371/journal.pone.0106050 25153994 PMC4143352

[B197] Williams D. R. Holton J. L. Strand C. Pittman A. de Silva R. Lees A. J. (2007). Pathological tau burden and distribution distinguishes progressive supranuclear palsy-parkinsonism from Richardson’s syndrome. Brain 130 (Pt 6), 1566–1576. 10.1093/brain/awm104 17525140

[B198] Wills J. Credle J. Haggerty T. Lee J. H. Oaks A. W. Sidhu A. (2011). Tauopathic changes in the striatum of A53T alpha-synuclein mutant mouse model of Parkinson’s disease. PLoS One 6 (3), e17953. 10.1371/journal.pone.0017953 21445308 PMC3061878

[B199] Wills J. Jones J. Haggerty T. Duka V. Joyce J. N. Sidhu A. (2010). Elevated tauopathy and alpha-synuclein pathology in postmortem Parkinson’s disease brains with and without dementia. Exp. Neurol. 225 (1), 210–218. 10.1016/j.expneurol.2010.06.017 20599975 PMC2922478

[B200] Wu C. Ma G. Li J. Zheng K. Dang Y. Shi X. (2013). *In vivo* cell tracking *via* 18F-fluorodeoxyglucose labeling: a review of the preclinical and clinical applications in cell-based diagnosis and therapy. Clin. Imaging 37 (1), 28–36. 10.1016/j.clinimag.2012.02.023 23206605

[B201] Wyss-Coray T. Mucke L. (2002). Inflammation in neurodegenerative disease—a double-edged sword. Neuron 35 (3), 419–432. 10.1016/S0896-6273(02)00794-8 12165466

[B202] Yoshida M. (2014). Astrocytic inclusions in progressive supranuclear palsy and corticobasal degeneration. Neuropathology 34 (6), 555–570. 10.1111/neup.12143 25124031

[B203] Yoshiyama Y. Higuchi M. Zhang B. Huang S. M. Iwata N. Saido T. C. (2007). Synapse loss and microglial activation precede tangles in a P301S tauopathy mouse model. Neuron 53 (3), 337–351. 10.1016/j.neuron.2007.01.010 17270732

[B204] Yu D. M. Zhao L. Y. Yang Z. Y. Chang S. Y. Yu W. T. Fang H. Y. (2016). Comparison of undernutrition prevalence of children under 5 years in China between 2002 and 2013. Biomed. Environ. Sci. 29 (3), 165–176. 10.3967/bes2016.021 27109127

[B205] Zarrouk A. Debbabi M. Bezine M. Karym E. M. Badreddine A. Rouaud O. (2018). Lipid biomarkers in Alzheimer’s disease. Curr. Alzheimer Res. 15 (4), 303–312. 10.2174/1567205014666170505101426 28474568

[B206] Zhang J. (2015). Mapping neuroinflammation in frontotemporal dementia with molecular PET imaging. J. Neuroinflammation 12, 108. 10.1186/s12974-015-0236-5 26022249 PMC4451729

[B207] Zverova M. Kitzlerova E. Fisar Z. Jirak R. Hroudova J. Benakova H. (2018). Interplay between the APOE genotype and possible plasma biomarkers in Alzheimer’s disease. Curr. Alzheimer Res. 15 (10), 938–950. 10.2174/1567205015666180601090533 29852871

